# The C-terminal tail of ribosomal protein Rps15 is engaged in cytoplasmic pre-40S maturation

**DOI:** 10.1080/15476286.2022.2064073

**Published:** 2022-04-19

**Authors:** Ingrid Rössler, Sarah Weigl, José Fernández-Fernández, Sara Martín-Villanueva, Daniela Strauss, Ed Hurt, Jesús de la Cruz, Brigitte Pertschy

**Affiliations:** a Institute of Molecular Biosciences, University of Graz, Graz, Austria; b BioTechMed-Graz, Graz, Austria; c Instituto de Biomedicina de Sevilla, Hospital Universitario Virgen del Rocío/CSIC/Universidad de Sevilla, Seville, Spain; d Departamento de Genética, Facultad de Biología, Universidad de Sevilla, Seville, Spain; e Biochemistry Center BZH, Heidelberg University, Heidelberg, Germany

**Keywords:** Ribosome biogenesis, 40S ribosomal subunit, ribosomal protein, rps15/uS19, yeast, chronic lymphocytic leukaemia, CLL

## Abstract

The small ribosomal subunit protein Rps15/uS19 is involved in early nucleolar ribosome biogenesis and subsequent nuclear export of pre-40S particles to the cytoplasm. In addition, the C-terminal tail of Rps15 was suggested to play a role in mature ribosomes, namely during translation elongation. Here, we show that Rps15 not only functions in nucleolar ribosome assembly but also in cytoplasmic pre-40S maturation, which is indicated by a strong genetic interaction between Rps15 and the 40S assembly factor Ltv1. Specifically, mutations either in the globular or C-terminal domain of Rps15 when combined with the non-essential *ltv1* null allele are lethal or display a strong growth defect. However, not only *rps15 ltv1* double mutants but also single *rps15* C-terminal deletion mutants exhibit an accumulation of the 20S pre-rRNA in the cytoplasm, indicative of a cytoplasmic pre-40S maturation defect. Since in pre-40S particles, the C-terminal tail of Rps15 is positioned between assembly factors Rio2 and Tsr1, we further tested whether Tsr1 is genetically linked to Rps15, which indeed could be demonstrated. Thus, the integrity of the Rps15 C-terminal tail plays an important role during late pre-40S maturation, perhaps in a quality control step to ensure that only 40S ribosomal subunits with functional Rps15 C-terminal tail can efficiently enter translation. As mutations in the C-terminal tail of human RPS15 have been observed in connection with chronic lymphocytic leukaemia, it is possible that apart from defects in translation, an impaired late pre-40S maturation step in the cytoplasm could also be a reason for this disease.

## Introduction

Eukaryotic ribosome assembly is a highly complex, hierarchical process that is initiated in the nucleolus upon transcription of a large ribosomal RNA precursor (35S pre-rRNA in yeast) and a pre-5S RNA accompanied by co-transcriptional association of the first ribosome assembly factors and ribosomal proteins (r-proteins). The resulting initial pre-ribosomal particles (90S particles or also called SSU processomes) undergo an intricate maturation pathway, involving more than 200 different assembly factors while separating into precursors for the small 40S and large 60S ribosomal subunits (r-subunits) and transiting to the nucleoplasm and finally to the cytoplasm. Seminal cryo-EM studies of yeast and human pre-ribosomal particles have shed light on the structures of intermediates along this ribosome maturation path, revealing that besides rRNA processing (summarized in Figure S1), also massive rRNA folding and restructuring events take place until rRNAs and r-proteins become compacted into functional mature r-subunits [1–3].

Upon endonucleolytic cleavage of the 35S pre-rRNA at processing site A_2_ and stepwise release of several structural modules of 90S particles in the nucleolus, the first pre-40S particles emerge [4,5]. These pre-40S particles contain the 20S pre-rRNA, a 3’-extended version of the mature 18S rRNA, as well as already most 40S r-proteins and, in yeast, nine stably bound assembly factors – Rrp12, Nob1, Pno1, Dim1, Tsr1, Rio2, Hrr25, Enp1 and Ltv1 [6]. These pre-40S particles are quickly exported into the cytoplasm by a still poorly understood export mechanism. Several proteins have been suggested to participate in pre-40S export in yeast, among them distinct putative export receptors (Crm1/Xpo1, Rrp12 and Mex67), putative adaptors for export receptor Crm1 (Ltv1, Rio2) and putative auxiliary proteins such as Slx9 as well as the r-protein S15 (Rps15/uS19^1^) [7–14].

Freshly exported pre-40S particles are not engaged immediately in translation as important functional sites are blocked by assembly factors [15]. In particular, Dim1, Rio2 and Tsr1 are positioned at the inter-subunit side, and prevent association of 60S and translation initiation factors [16–18]. In the course of cytoplasmic pre-40S maturation, all pre-40S assembly factors are successively released, while, concomitantly, r-proteins which are not yet assembled at their final position are stably accommodated, and in addition, the last missing r-proteins are incorporated into pre-40S particles. For instance, the kinase Hrr25 phosphorylates and releases Ltv1 promoting the stable accommodation of r-protein Rps3/uS3 [19–21]. This maturation event is coordinated with the ATP-hydrolysis-triggered self-release of the ATPase Rio2 [22–24]. Obviously, a recurring theme in ribosome assembly is quality control, in which assembly factors bind to functionally important sites of the nascent r-subunits and probe for correct structure and/or function. In yeast, these maturation steps are believed to occur in a ‘translation-like cycle’, in which an immature pre-40S particle is joined *via* eukaryotic initiation factor 5B (eIF5B) with an apparently mature 60S subunit [25–27]. Only when this putative ‘translation test-drive’ is successful, the final maturation steps are triggered. The hallmark event of these final 40S maturation steps is the processing of the 20S pre-rRNA into the mature 18S rRNA by the endonuclease Nob1, involving the release of the Nob1-inhibitor Pno1, and finally Nob1, by Rio1 ATPase [28–30].

A key prerequisite for the progression of ribosome biogenesis is the correct and timely binding of r-proteins, as reflected by the fact that the absence of almost any of the 79 yeast r-proteins lead to a specific maturation defect, either in nucleolar ribosome biogenesis steps, in r-subunit export, or maturation steps after export [31–33]. Apart from that, many r-proteins have additional functions that could only be revealed by specific point mutants. The advantage is that such mutant r-protein variants can still assemble onto pre-ribosomal particles and allow maturation to proceed but are disturbed in one of their specific functions. Two well-characterized examples are Rps14/uS11 and Rps5/uS7: while their depletion leads to nucleolar pre-rRNA processing defects [31], Rps14 and Rps5 variants carrying mutations or deletions in the C-terminal unstructured extensions of the proteins cause defects in cytoplasmic 20S pre-rRNA processing, indicating an additional function of these proteins in cytoplasmic pre-40S maturation [34,35].

We are interested in understanding the function of Rps15, an r-protein of the small 40S subunit, which is highly conserved among eukaryotes (Figure S2A). Both in yeast and human cells, depletion of Rps15 was shown to cause retention of pre-40S particles in the nucleus, suggesting that Rps15 assembly onto pre-40S particles is essential for the export of these particles [11,12,31]. Additionally, an earlier, nucleolar function of Rps15 was revealed by a synthetic lethality study with a thermosensitive *rps15-1* mutant, uncovering genetic links of *RPS15* with *NHP2, UTP15, SLX9*, and *BUD23*. Combined mutations of *NHP2, UTP15* or *SLX9* with *rps15-1* led to nucleolar accumulation of pre-40S particles and early pre-rRNA processing defects [36]. In contrast, the *rps15-1 Δbud23* double mutant primarily showed nucleoplasmic pre-40S accumulation, and, therefore, defects in nuclear export of pre-40S particles [36].

Besides these functions in 40S subunit biogenesis, Rps15 also fulfils important roles in the mature ribosome. The globular domain of Rps15 is engaged in the B1a inter-subunit bridge by interacting with helix H38 of the 25S rRNA [37,38]. In turn, the largely unstructured C-terminal tail of Rps15 reaches to the decoding site of the 40S subunit, thereby playing an important role in translation elongation [39,40]. Recently, the very C-terminal residues of Rps15 could be visualized in a mature human 80S ribosome, revealing its flexibility during translation [41]. In the post-decoding pre-translocation state of translation elongation, the Rps15 C-terminus interacts with both A- and P-site tRNAs and the mRNA in the decoding site [40]. Based on these structural data, the Rps15-C-terminal domain was suggested to be engaged in the efficient accommodation of tRNAs at the A-site. Notably, mutations in the Rps15 C-terminal tail have been found with a high frequency in patients with relapsing chronic lymphocytic leukaemia (CLL), further substantiating the important function of the Rps15 C-terminus [42–44].

In this study, we identified a specific *rps15* mutant, carrying an F138S exchange in the C-terminal tail of the Rps15 r-protein, which shows a synthetically enhanced growth defect together with a Δ*ltv1* chromosomal deletion. A second mutation, F102L, positioned in the region from where the C-terminal tail emerges, further enhanced this defect. In pre-40S particles, both F138 and F102 residues are positioned in close proximity to 40S assembly factor Tsr1. Consequently, *tsr1* mutants genetically interact with these different *rps15* mutants. Further investigation of these as well as other mutations in Rps15 indicated that, in contrast to the Rps15 depletion, nucleo-cytoplasmic export of pre-40S particles is not impaired, but the final cytoplasmic pre-40S maturation is disturbed. We conclude that the Rps15 C-terminal tail is not only needed for correct translation but already required during the final steps of 40S subunit synthesis.

## Results

### A synthetic lethality screen links Rps15 to 40S assembly factor Ltv1

**Figure 1.**
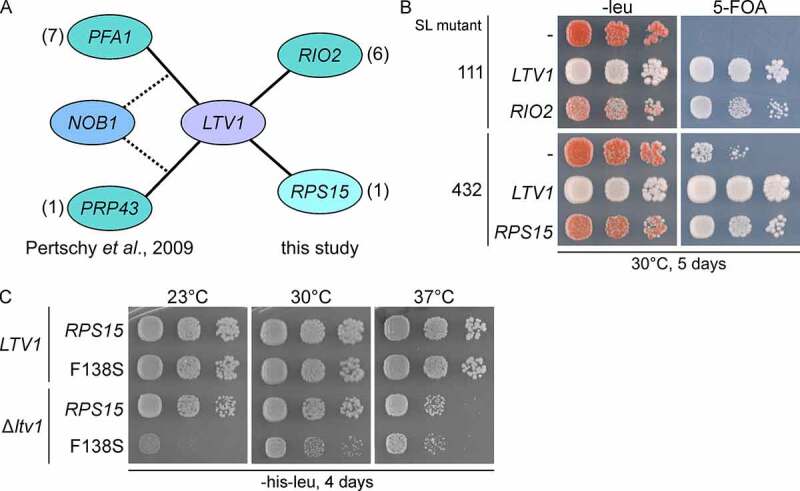
*LTV1* is genetically linked to *RPS15*. (A) Schematic overview of the genetic interactions uncovered in a synthetic lethality (SL) screen with a Δ*ltv1* strain. Genes displayed on the left side were identified in a previous study [30], while genes identified in the current study are shown on the right side. (B) Mutants #SL111 and #SL432 isolated in the SL screen were transformed with *LEU2*-plasmids carrying the indicated genes or with an empty plasmid (-); transformants were spotted in serial 10-fold dilution steps onto SDC-leu plates (-leu) as well as plates containing 5-fluoroorotic acid (5-FOA), and incubated at 30°C for 5 days. Red colony colour on SDC−leu and slow growth on 5-FOA plates indicate a synthetic growth defect. Red/white sectoring on SDC-leu and cell growth on 5-FOA plates indicate complementation of the synthetic enhancement phenotype. (C) Temperature dependence of the observed genetic interactions. Strains carrying the indicated wild-type and mutant *RPS15* alleles on *LEU2*-plasmids plus *HIS*3-empty plasmid (Δ*ltv1*) or *LTV1* wild-type allele on a *HIS3*-plasmid were spotted onto SDC-his -leu (-his -leu) plates to select for the transformed plasmids and incubated at 23°C, 30°C, and 37°C for 4 days.

Previously, we have performed a synthetic lethality (SL) screen with a deletion mutant of the non-essential 40S subunit assembly factor Ltv1, revealing its functional connection to RNA helicase Prp43, and its G-patch cofactor Pfa1 [30]. The synthetically enhanced growth and 20S pre-rRNA processing defects observed upon *LTV1* deletion in *prp43* or *pfa1* mutants were high-copy-suppressed by *NOB1* (Figure 1A and [30]). These results indicated a role of Prp43 and its cofactor Pfa1 in promoting 20S pre-rRNA processing by the endonuclease Nob1.

To identify additional players in late pre-40S maturation functionally connected to Ltv1, we set out to investigate seven of the so far uncharacterized mutants found in this SL screen. Considering the previous observations of a synthetically sick phenotype upon combination of certain *rio2* mutants with the null or point-mutant *ltv1* alleles [23,24], we transformed the remaining mutants with a *RIO2* containing plasmid and scored for complementation of synthetic growth defects, as evaluated by a white or red/white sectoring colony colour and the ability to grow on 5-FOA containing plates (see Methods section for details). Indeed, six of these seven mutants could be complemented by *RIO2* (Fig. 1(A,B), exemplarily shown for mutant #111). This strong genetic link between *LTV1* and *RIO2* is also in agreement with their joint action during cytoplasmic pre-40S maturation [24].

One remaining mutant (#432) was neither complemented by *RIO2, PRP43* and *PFA1* nor suppressed by *NOB1*. To identify the mutation leading to synthetic enhancement in this strain, we transformed a yeast genomic library into the mutant and screened for transformants re-establishing the red/white sectoring phenotype, and hence for the ability to lose the *LTV1* containing plasmid.

Using this strategy, we found that the original SL mutant #432 was complemented by the *RPS15* gene (Figure 1(A,B)). Sequencing of the chromosomal copy of this gene confirmed a mutation, leading to a phenylalanine to serine exchange (F138S) in the C-terminal unstructured tail of the Rps15 protein (position indicated in Figures S2B and 3A).

To avoid interference by other mutations arising in the strain generated by UV mutagenesis, we constructed a strain in which the chromosomal copy of *RPS15* was deleted and complemented by a low-copy plasmid carrying the *rps15-*F138S allele; then, the growth of this mutant was compared to that of the same strain but carrying the *RPS15* wild-type (WT) plasmid. While the *rps15-*F138S mutant did not display any growth defects by itself at any tested temperature (23°C, 30°C or 37°C), it strongly enhanced the growth defect of a Δ*ltv1* strain, in particular at low temperature, indicating a genetic interaction between *LTV1* and the *rps15*-F138S allele (Fig. 1C).

To exclude that this genetic interaction is caused by a reduced level of the Rps15-F138S compared to the wild-type protein, we chromosomally fused the sequence encoding an N-terminal 2xHA-tag to *RPS15* and *rps15-*F138S in the presence and absence of *LTV1*, respectively. Rps15 protein levels, detected with an α-HA-antibody, were apparently similar in the wild-type and the *rps15*-F138S mutant strain at any of the temperatures tested (Figure S3A). Based on these results, we conclude that there is a specific functional link between Ltv1 and the C-terminal domain of Rps15. As the chromosomally integrated constructs, however, displayed growth defects due to the N-terminal 2xHA-tag (Figure S3B), we performed all subsequent experiments using untagged Rps15 and variants thereof, encoded from a low-copy plasmid in the background of the knockout of the chromosomal *RPS15* copy.

### The rps15-F138S mutation enhances the 40S maturation defect of the Δltv1 deletion

**Figure 2.**
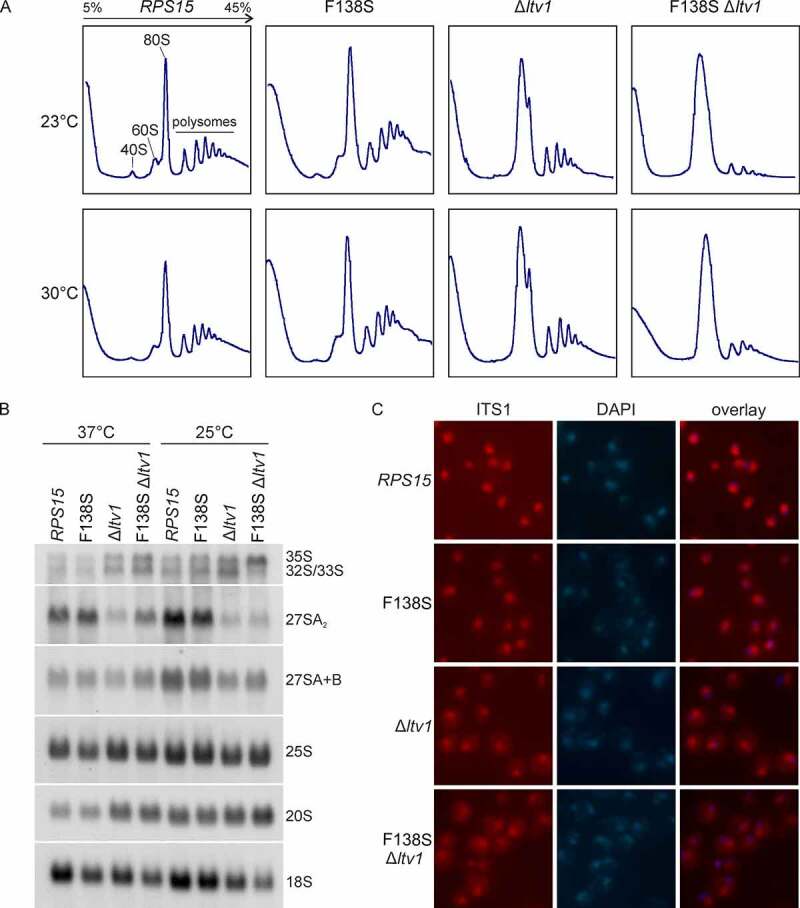
40S maturation defect of the Δ*ltv1* strain is enhanced by the *rps15*-F138S allele. (A) Yeast cells of the Δ*rps15* and Δ*rps15* Δ*ltv1* strains carrying a *LEU2*-plasmid encoding wild-type *RPS15* or *rps15*-F138S were grown in liquid SDC-leu medium at 23°C or 30°C, respectively. After addition of cycloheximide and inhibition of translation, cells were lysed and polysome profiles recorded. Peaks corresponding to the free 40S and 60S subunits, 80S ribosomes and polysomes are indicated in the profile of the wild-type strain at 23°C. (B) *rps15*-F138S enhances the 20S pre-rRNA accumulation occurring in a Δ*ltv1* strain. For northern blotting, cells were grown in liquid SDC-leu at the indicated temperatures. For pre- and mature rRNA detection, the indicated probes were used (the yeast pre-rRNA processing pathway is shown in Figure S1). (C) The mutant *rps15*-F138S shows no pre-40S export defect but an impairment in cytoplasmic 20S pre-rRNA processing. For fluorescence *in situ* hybridization (FISH), cells were grown in liquid SDC-leu at 30°C. After cell fixation, spheroplasts were incubated with a probe specific to the D/A_2_ segment of the ITS1 region to detect 20S pre-rRNA and nuclei were stained with DAPI.

Considering the genetic link of the *rps15-*F138S mutant to the late 40S assembly factor Ltv1, we speculated that the Rps15 C-terminal tail may be important for 40S subunit maturation. Polysome profiling indicated that the *rps15-*F138S mutant showed a mild 40S subunit shortage, as concluded from the slightly increased free 60S subunit peak (Figure 2A). The Δ*ltv1* mutant showed a decreased free 40S peak and a strongly increased free 60S peak, as observed previously [30]. An increased free 60S peak is characteristic for a 40S synthesis defect, as a deficit of available 40S subunits for subunit joining is leading to a consequent accumulation of free 60S subunits. Importantly, the *rps15-*F138S mutation clearly enhanced the 40S subunit synthesis defect of the Δ*ltv1* mutant, as obvious from the absence of a free 40S subunit peak, and a massive increase of a combined 60S subunit/80S ribosome peak compared to the Δ*ltv1* mutant alone. Moreover, polysomes were drastically reduced in the *rps15-*F138S Δ*ltv1* strain at 30°C, and even more so at 23°C, indicating that the 40S subunit synthesis defect results in reduced translation elongation (Figure 2A).

To determine the underlying maturation defects more precisely, we performed northern blotting and detected several intermediates of the pre-rRNA processing pathway (see also the schematic overview of the pathway in Figure S1). While the *rps15-*F138S mutant alone did not display any apparent defects, this mutation enhanced the defects observed for the Δ*ltv1* strain, especially at 25°C, leading to a slight additional accumulation of 20S pre-rRNA as compared to Δ*ltv1* alone and, even more noticeable, a reduction of mature 18S rRNA levels. Moreover, possibly as a secondary effect, the 35S pre-rRNA accumulated in this double mutant at low temperature (Figure 2B).

Both the Δ*ltv1* deletion and Rps15 depletion were previously observed to result in 40S subunit export defects [12,13,31]. To assess if the cause for the strong growth defect of the *rps15-*F138S Δ*ltv1* double mutant is a pre-40S export defect or a defect prior to pre-40S export, we examined the localization of the 40S subunit reporter Rps3-GFP (uS3-GFP) in this mutant by fluorescence microscopy (Figure S4). No significant nuclear accumulation of Rps3-GFP was observed in the *rps15-*F138S Δ*ltv1* double mutant as compared to Δ*ltv1* alone, pointing towards a later, mainly cytoplasmic defect of the double mutant. Fluorescence *in situ* hybridization (FISH) using a probe specific to the D/A_2_ segment of ITS1 further indicated that 20S pre-rRNA synthesized in the *rps15-*F138S Δ*ltv1* mutant can be exported into the cytoplasm, where, similar to the Δ*ltv1* mutant, increased amounts of 20S pre-rRNA are detected; thus, its cytoplasmic processing is impaired (Figure 2C). We conclude that besides the previously reported function of Rps15 in 40S subunit export, Rps15 is also required for cytoplasmic steps of pre-40S maturation.

### Expanding the genetic network between Rps15 and late 40S maturation factors

**Figure 3.**
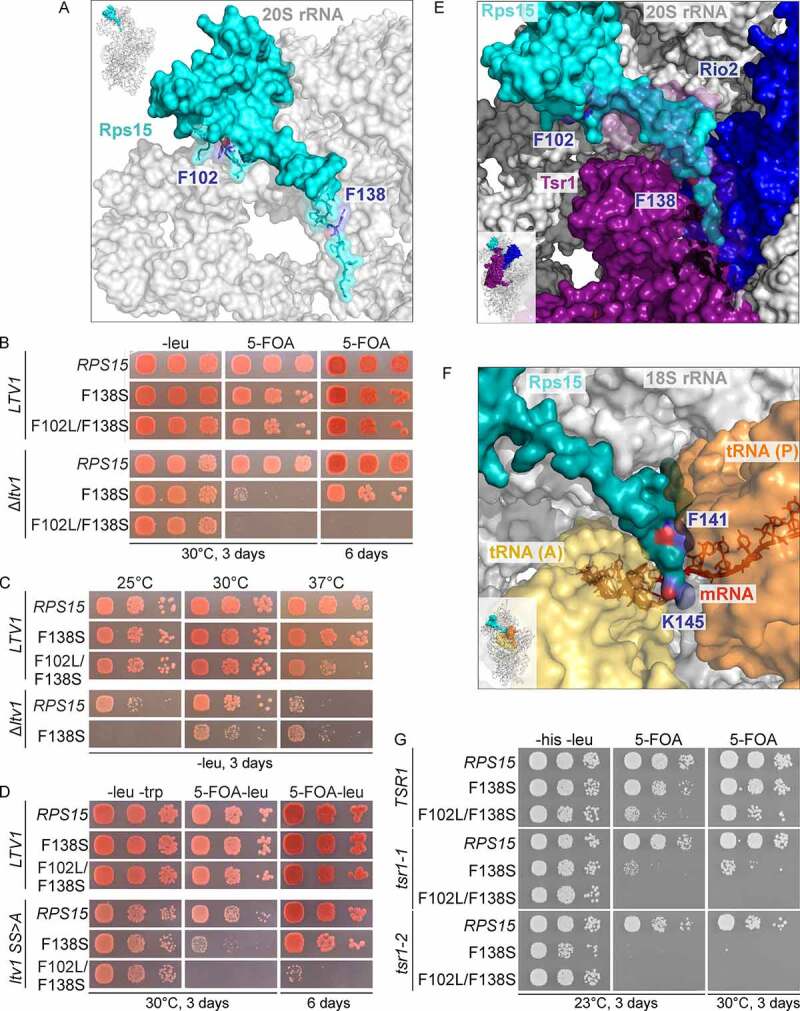
The *rps15*-F102L/F138S double mutation is genetically linked to *LTV1* and *TSR1*. (A) The amino acid exchange F138S is located at the C-terminus of Rps15, whereas the second mutation F102L is positioned in the globular domain at which the C-terminus emerges ([45], PDB 6Y7C). (B) Genetic interaction between the Δ*ltv1* null allele and the double mutant *rps15-*F102L/F138S. The Δ*rps15* and Δ*rps15* Δ*ltv1* strains carrying the wild-type plasmid *URA3-RPS15* as well as a *LEU2*-plasmid encoding *RPS15, rps15*-F138S or *rps15*-F102L/F138S, respectively, were spotted onto SDC-leu plates and plates containing 5-FOA and were incubated at 30°C for 3 and 6 days. (C) In the Δ*ltv1* strain, the *RPS15*-mutation F138S leads to cold sensitivity. The shuffled Δ*rps15* and Δ*rps15* Δ*ltv1* strains, shown in B, containing only a *LEU2*-plasmid encoding *RPS15, rps15*-F138S or *rps15*-F102L/F138S were spotted onto SDC-leu plates and incubated for 3 days at the indicated temperatures. (D) Genetic interaction between the phosphorylation deficient *ltv1* mutant (*ltv1-*SS>A) and *RPS15*. The Δ*rps15* Δ*ltv1* cells carrying *URA3-RPS15* and *LEU2-LTV1* or *LEU2-ltv1-*SS>A as well as a *TRP1*-plasmid with *RPS15, rps15*-F138S or *rps15*-F102L/F138S, respectively, were spotted onto SDC-leu-trp plates (-leu -trp) and plates containing 5-FOA and lacking leucine (5-FOA-leu) and were incubated at 30°C for 3 and 6 days. (E) Rps15 is in contact with Rio2 and Tsr1 in the structure of the pre-40S particle ([45], PDB 6Y7C). Rps15 is coloured in teal, Rio2 in blue and Tsr1 in purple. Parts of Tsr1 are more transparent to reveal the C-terminus of Rps15 behind Tsr1. (F) The Rps15 C-terminal tail is positioned between the A- and P-site tRNAs in the post-decoding pre-translocation translating human ribosome ([41], PDB 6Y0G). The indicated position K145 (C-terminal residue of human RPS15) corresponds to yeast K142, the indicated F141 corresponds to yeast F138. (G) Genetic interaction between *TSR1* and *RPS15*. The double knockout strain Δ*rps15* Δ*tsr1* carrying *URA3-RPS15* and *URA3-TSR1* as well as combinations of *LEU2-*plasmids with *TSR1, tsr1-1, tsr1-2* and the *HIS3*-plasmids with *RPS15, rps15* F138S or *rps15* F102L/F138S were spotted onto SDC-his-leu plates (-his -leu) and plates containing 5-FOA and were incubated at 23°C or 30°C for 3 days.

Given the link of the *rps15*-F138S mutation to *LTV1*, we wanted to further explore the connections of *RPS15* to late 40S maturation. In addition to the *rps15-*F138S mutant, we included a second mutant into these analyses, which was erroneously generated during PCR amplification of the *rps15-*F138S allele during cloning. The resulting mutant allele contains a phenylalanine to leucine exchange (F102L) in addition to the F138S exchange. Whereas F138 is positioned only five amino acids from the C-terminal end of Rps15, F102 is positioned in the globular domain in the region from where this C-terminal tail emerges (positions indicated in Figure 3(A)). Notably, the *rps15-*F102L/F138S mutant revealed a slight growth defect at 37°C on its own and was lethal when combined with a Δ*ltv1* null mutant (Figure 3(B,C)).

Previous studies suggested that *LTV1* deletion results in a mixed phenotype, showing both pre-40S export and cytoplasmic pre-40S maturation defects (see, for example, [30]). To further validate our hypothesis that Rps15 has a function in cytoplasmic 40S maturation, we examined *ltv1* mutants with no export but only cytoplasmic defects for genetic interactions with *rps15* mutants. We have previously generated several *ltv1* mutants in which the phosphorylation of the corresponding Ltv1 protein by Hrr25 is reduced, thus, resulting in an inhibition of the release of Ltv1 in the cytoplasm and consequently an exclusively cytoplasmic pre-40S maturation defect; these mutants include the *ltv1*-S339A/S342A mutant [20]. Indeed, this phosphorylation deficient *ltv1* mutant also showed a genetic interaction with both *rps15* alleles of this study, with the *rps15-*F102L/F138S allele showing an almost lethal phenotype in combination with *ltv1*-S339A/S342A (abbreviated as *ltv1*-SS>A; (Figure. 3(D)) and **S5A**).

Given the genetic links of *LTV1* to *PFA1* ^30^ and *RIO2* ^23^ (Figure. 1(A)), we next addressed if *RPS15* also genetically interacts with these two genes. However, no genetic interaction was observed between our *rps15* mutant variants and a Δ*pfa1* null allele or the *rio2-1* point-mutant (Figure S5B and S5C).

We then inspected recent pre-40S cryo-EM structures, to identify assembly factors which might function together with Rps15. Interestingly, while the C-terminal tail of Rps15 is positioned between the A- and P-site tRNAs in the post-decoding pre-translocation state in translation (Figure 3(F)) [41], this tail is positioned between 40S assembly factors Rio2 and Tsr1 in pre-40S particles (Figure 3(E)) [18,45]. Moreover, domain II of Tsr1 reaches close to the region in which the F102L exchange in the Rps15 mutant variant is positioned. Therefore, we tested for a genetic interaction with the essential *TSR1* gene. To this end, we generated *tsr1* mutants by random PCR mutagenesis. Two thermo-sensitive *tsr1* alleles were obtained, termed *tsr1-1* and *tsr1-2*, leading to three (E588D, Y604C and T704A) and eight (F220L, L239F, R473 G, E481G, F596Y, F710L, T716A, F759L) exchanges in the amino acid sequence of the Tsr1 protein, respectively. Interestingly, both *tsr1* mutant alleles caused synthetic lethality in combination with the *rps15*-F102L/F138S mutation and a synthetically enhanced defect in combination with the *rps15*-F138S mutation (Figure 3(G)) and S5D. These results may be an indication that the contact between Rps15 and Tsr1 is important for cytoplasmic pre-40S subunit maturation.

### The C-terminal tail of Rps15 functions in late 40S subunit maturation

The so far obtained *rps15* mutants display no (*rps15*-F138S) or only mild (*rps15*-F102L/F138S) defects by themselves, and their connection to late 40S maturation becomes only obvious when combined with other mutations (e.g. *ltv1* or *tsr1*). Therefore, we next aimed to generate *rps15* mutants displaying late 40S maturation defects on their own.

**Figure 4.**
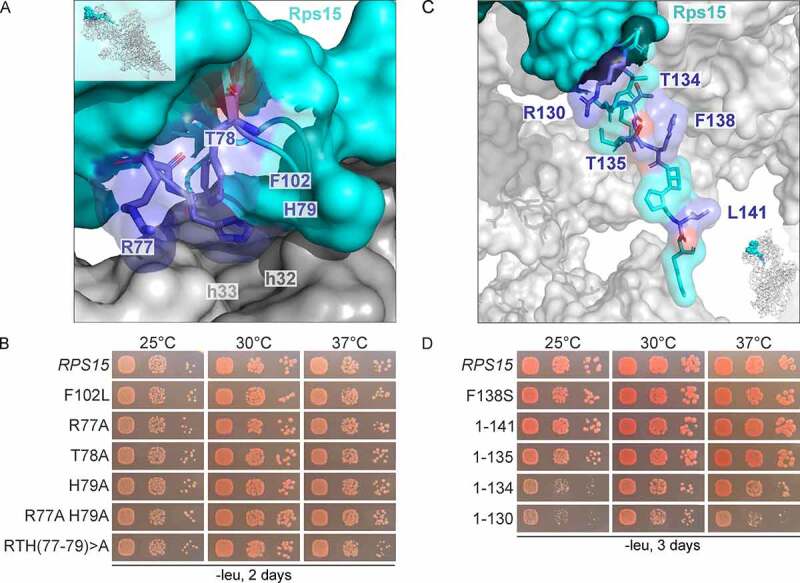
C-terminal Rps15-truncation results in growth defects. (A) The F102L exchange in Rps15 is in close proximity to the amino acids R77 and H79. In the pre-40S particle ([45], PDB 6Y7C), R77 is in contact with helix h33 of the 18S rRNA, and H79 is in contact with helix h32. The third amino acid in this globular region of Rps15, that was exchanged in this study, is threonine 78 (T78). (B) The triple mutation RTH(77–79)>A of *RPS15* leads to a slight growth defect at high temperatures. The shuffled Δ*rps15* strains, containing a *LEU2-RPS15* plasmid or *LEU2-rps15* variant as indicated were spotted onto SDC-leu plates (-leu) and incubated for 2 days at the indicated temperatures. (C) C-terminal deletions, investigated in this study, are indicated in the pre-40S particle ([45], PDB 6Y7C). (D) *RPS15* C-terminal deletions lead to growth defects. The shuffled Δ*rps15* strains, containing only a *LEU2-RPS15* plasmid or C-terminal deleted *rps15* variants as indicated were spotted onto SDC-leu plates and incubated for 3 days at the indicated temperatures.

For this purpose, we created further mutations in proximity to F102 and F138. Residue F102 does not directly contact RNA nor other proteins; however, in the mature 40S and pre-40S structures, it is in close proximity to arginine 77 (R77) and histidine 79 (H79), which are in direct contact with 18S rRNA helices h32 and h33 (Figure 4(A) [37,45]. Therefore, we speculated that the F102L mutation may affect the positioning of these residues, consequently leading to altered interactions of Rps15 with rRNA. To further evaluate the importance of these residues, we mutated R77 and H79, as well as the residue in between, threonine 78 (T78), alone and in combinations (Fig. 4B). However, although most of them (except the R77A mutant) were genetically linked to *LTV1* (Figure S6A and S6B), many of these mutants did not display a growth defect on their own, except for the triple mutant *rps15*-R77A/T78A/H79A (further referred to as *rps15*-RTH(77–79)>A), which showed a slight growth defect at 37°C (Fig. 4B).

Next, as F138 is very close to the C-terminal end of Rps15, we generated stepwise C-terminal truncations (depicted in Fig. 4C) and compared the growth of the different mutants to that of the F138S mutant. Removal of the last seven amino acids (*rps15*-1-135) led to a slight growth defect, deletion of the last eight residues (*rps15*-1-134) to a strong growth defect, which became even more severe when the last 12 (*rps15*-1-130) amino acids were removed (Fig. 4D). Notably, all tested C-terminally truncated Rps15 variants, including a variant lacking only the very last amino acid (lysine, K142), led to a synthetic enhanced defect (*rps15*-1-141) or even a synthetic lethal phenotype (all other C-terminally truncated mutants) in combination with the Δ*ltv1* null allele (Figure S6C and S6D).

**Figure 5.**
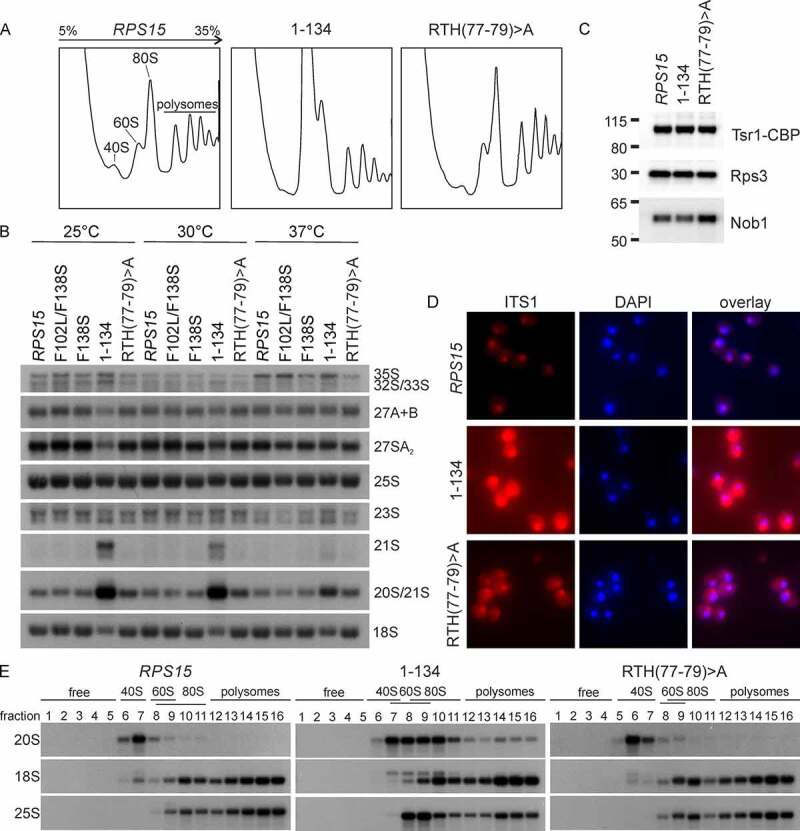
The C-terminus of *RPS15* functions in late pre-40S maturation. (A) Δ*rps15* cells carrying the *LEU2*-plasmid encoding the wild-type *RPS15*, *rps15-*1-134 or *rps15-*RTH(77–79)>A, respectively, were grown in liquid SDC-leu medium at 30°C. After inhibition of translation by cycloheximide, cells were lysed and polysome profiles were recorded. Peaks corresponding to the 40S and 60S subunit, 80S ribosomes and polysomes are indicated in the profile of the wild-type strain. (B) For northern blotting, *rps15* deletion strains carrying *LEU2*-plasmids with wild-type *RPS15* or the indicated *rps15* variants were grown in liquid SDC-leu medium at 30°C to an OD_600_ of 0.1–0.2 and shifted for 3 h to 25°C, 30°C or 37°C, respectively. Blots were probed as indicated in the methods section (the yeast pre-rRNA processing pathway is shown in Figure S1). (C) Eluates from TAP purification of Tsr1-particles from the wild-type *RPS15* or *rps15-*1-134 or *rps15-*RTH(77–79)>A mutant backgrounds were analysed via SDS-PAGE, followed by western blotting. Tsr1-CBP was detected via α-CBP antibody; Rps3 and Nob1 were detected with antibodies specific to the respective proteins. (D) The *rps15-*1-134 and *rps15-*RTH(77–79)>A mutants show cytoplasmic 20S pre-rRNA accumulation. For fluorescence *in situ* hybridization (FISH), cells were grown in liquid SDC-leu at 30°C. After cell fixation, spheroplasts were incubated with a probe specific to the D/A_2_ segment of the ITS1 region to detect 20S pre-rRNA and nuclei were stained with DAPI. (E) After fractionation of polysomes from the *RPS15* strain and *rps15-*1-134 or *rps15-*RTH(77–79)>A, respectively, northern blots were performed. Blots were probed as described in the methods section.

To address whether the novel *rps15* mutants also display late 40S maturation defects on their own, we investigated the phenotypes of the newly generated *rps15*-RTH(77–79)>A and *rps15-*1-134 mutants. Polysome profiles showed a slight (*rps15*-RTH(77–79)>A) or severe (*rps15*-1-134) increase in the free 60S peak, together with a reduction of the free 40S peak, indicating a 40S subunit maturation defect in these mutants (Fig. 5A).

To detect potential rRNA processing defects in these new *rps15* mutants, we performed northern blotting and included the *rps15*-F138S and *rps15*-F102L/F138S mutants in the analysis for comparison. Indeed, we observed a slight (*rps15*-RTH(77–79)>A) or strong (*rps15*-1-134) accumulation of 20S pre-rRNA, which was, in the case of the *rps15*-1-134 mutant also accompanied by a reduction of mature 18S rRNA, especially at low temperatures. In addition, in the *rps15*-1-134 mutant, a 21S pre-rRNA was also detected at 30°C and to a higher extent at 25°C (Fig. 5B). This aberrant precursor arises when cleavage of the 32S pre-rRNA intermediate at site A_2_ is skipped and the precursor is cleaved at site A_3_ instead (see Figure S1). In line with this, a mild accumulation of 32S (and also 35S) pre-rRNA and a reduction of 27SA_2_ pre-rRNA was also observed under the same conditions. These data suggest that the *rps15*-1-134 mutant also shows a defect in early nucleolar ribosome biogenesis, next to the late defect in 20S pre-rRNA processing. As 20S pre-rRNA is cleaved by Nob1, a potential explanation for 20S pre-rRNA accumulation in the *rps15* mutants could be the failure to recruit Nob1 to pre-40S particles. However, Nob1 was present in late nuclear and cytoplasmic pre-40S particles purified from the *rps15*-1-134 and *rps15*-RTH(77–79)>A strains, indicating that a step after Nob1 recruitment has to be affected (Fig. 5C). In addition, to determine, in which cellular compartment the 20S pre-rRNA accumulates, we assessed the localization of 20S pre-rRNA in the mutants by FISH (Fig. 5D). While the *rps15*-RTH(77–79)>A mutant showed only slight cytoplasmic accumulation of 20S pre-rRNA, the C-terminal truncation mutant *rps15*-1-134 accumulated significant amounts of 20S pre-rRNA in the cytoplasm, indicating a strong cytoplasmic 40S maturation defect before site D is cleaved by Nob1.

Although cytoplasmic 20S pre-rRNA accumulation was the strongest phenotype observed in the two *rps15* mutants, the *rps15*-1-134 mutant also appeared to have an increased nuclear ITS1 signal (Fig. 5D) and showed some accumulation of nuclear pre-rRNAs, i.e. 21S, 23S, 32S and 35S pre-rRNAs (Fig. 5B). Therefore, we sought for further confirmation that the cytoplasmic 20S pre-rRNA accumulation is the main defect in these mutants. To this end, we scored for potential pre-40S export defects in these two *rps15* mutants, by examining the localization of the 40S subunit export reporter Rps3-GFP via fluorescence microscopy (Figure S7A). As a control, also the 60S subunit export reporter Rpl25-GFP was examined in these strains. Importantly, neither the *rps15*-RTH(77–79)>A nor the *rps15*-1-134 mutant showed an r-subunit export defect (Figure S7A), further supporting our model that they mainly display cytoplasmic pre-40S maturation defects. In our previous study, we observed that large amounts of the 20S pre-rRNA accumulating in Δ*ltv1* Δ*pfa1* as well as Δ*ltv1 prp43* double mutants are incorporated into polysomes [30], suggesting that in these mutants, 20S pre-rRNA containing 40S subunits can escape the quality control exerted by the 'translation-like cycle' and engage in translation. To address if such a phenotype is also observable in the *rps15*-1-134 and *rps15*-RTH(77–79)>A mutants, we analysed fractions collected from polysome profiles of these strains by northern blotting (Figure 5E). In the *rps15*-RTH(77–79)>A mutant, the 20S pre-rRNA sedimented with the 40S peak comparable to the wild-type strain. In contrast, the *rps15*-1-134 mutant showed small amounts of 20S pre-rRNA in polysomes; however, in contrast to the Δ*ltv1* Δ*pfa1* and Δ*ltv1 prp43* double mutants investigated previously [30], most of the 20S pre-rRNA signal was found in the 80S peak, suggesting that pre-40S particles from the *rps15*-1-134 mutant can form 80S ribosomes but engage only inefficiently in translation. Taken together, we conclude that the C-terminal domain of Rps15 is important for the final cytoplasmic pre-40S subunit maturation events prior to 20S pre-rRNA processing.

### The C-terminal domain of Rps15 acts together with the N-terminal extension of Rps31 in ensuring translational fidelity

Recent findings suggested an important function of the Rps15 C-domain in translational decoding [39,41], while our data indicate an additional role of Rps15 in late pre-40S maturation. These phenotypes are reminiscent of those described for r-protein Rps31/eS31, for which a dual role in late 40S maturation and decoding was also described [46–48], and a *∆rps31* (also known as *∆ubi3*) mutant was also found to be synthetic lethal with *∆ltv*1 [47].

**Figure 6.**
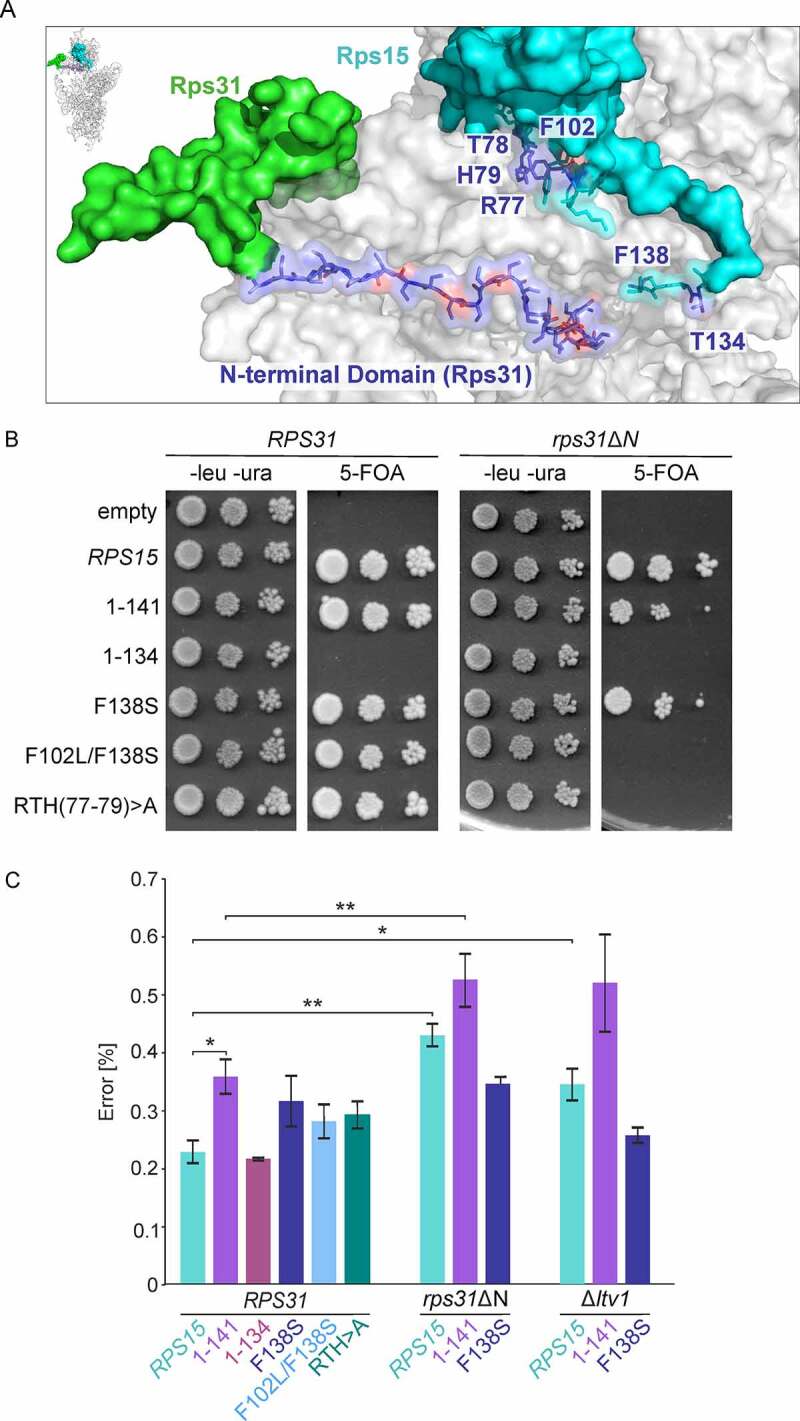
Rps31 and Rps15 are functionally linked and ensure translational fidelity. (A) The Rps15 C-terminal tail is in close proximity to the Rps31 N-terminal tail in mature 40S subunits (PDB 4V88 [37]). (B) Genetic interaction between *RPS15* and *RPS31*. A Δ*rps15 rps31*ΔN [*URA3-RPS15*] shuffle strain carrying the indicated wild-type and mutant alleles was spotted onto SDC-leu-ura plates (-leu -ura) and 5-FOA containing plates. The *rps15-*1-134 *rps31*ΔN, the *rps15*-F102L/F138S *rps31*ΔN and *rps15-*RTH(77–79)>A *rps31*ΔN mutants were inviable on 5-FOA, indicating synthetic lethality. (C) Measurement of misincorporation frequencies in the indicated mutants. Strains were transformed with *URA3*-plasmids pDB688 and pDB868 to measure misreading (Arg245(GGC) to His245(CAC)). Transformants were grown in liquid SDC-ura medium to mid-log phase at 30°C and then *Renilla* and firefly luciferase activity was measured. Assays were done in quadruplicate, and the data were expressed as the mean ± the standard deviation. The percentage of misreading was expressed as the firefly (H245R)/*Renilla* luciferase activity divided by the firefly (wild-type)/*Renilla* luciferase activity multiplied by 100. Significance levels were determined by Student’s t-test (*, p < 0.05).

Moreover, Rps31 is positioned at the beak of the 40S subunit and has an unstructured N-terminal extension, which reaches into close proximity to the C-terminal domain of Rps15 (Fig. 6A) [37]. Similar to C-terminal deletions of Rps15, the deletion of the N-terminal domain of Rps31 (*ubi3*Δ*N* allele, herein *rps31ΔN*) or complete deletion of the non-essential *RPS31* gene leads to late pre-40S maturation defects, including a huge 20S pre-rRNA accumulation in the cytoplasm. Moreover, in *ubi3Δ* cells, 20S pre-rRNA mainly co-sediments with 80S ribosomes, with small amounts of 20S pre-rRNA entering into polysomes [47], similar as we observed for the *rps15*-1-134 mutant. Besides, cells lacking Rps31 show an increased rate of amino acid misincorporation during translation, indicating that Rps31 ensures optimal translational fidelity [47]. In order to find out if Rps15 and Rps31 could cooperate in their functions, we first tested for a genetic interaction between *rps15* and *rps31* N-terminal mutants. Indeed, the *rps31*ΔN mutant was synthetically lethal in combination with the *rps15-*1-134, *rps15*-F102L/F138S and *rps15*-RTH(77–79)>A alleles (Fig. 6B). Moreover, *rps15*-F138S, as well as *rps15-*1-141 showed a mild synthetically enhanced growth defect in combination with the *rps31*ΔN allele (Figure S8).

To assess if these synthetic growth defects go along with an enhancement of the translational error rate upon combination of *rps15* and *rps31* mutations, we analysed amino acid misincorporation in the viable *rps15 rps31*ΔN double mutants (Fig. 6C). Additionally, we also analysed the *rps15* single mutants F138S, 1–141, 1–134, F102L/F102S and RTH(77–79)>A. To this end, we made use of a plasmid-borne tandem *Renilla* and firefly luciferase reporter system [49], where the CAC (His) codon at position 245, essential to form functional firefly luciferase, has been substituted by a CGC (Arg) codon, resulting in only residual firefly luciferase enzymatic activity. An increased firefly luciferase activity is indicative of a misreading event, leading to misincorporation of histidine at this critical position, and consequently to reconstitution of the enzymatic activity of the protein.

Our results indicated that the single *rps31∆N* and the *rps15*-1-141 mutation leads to a modest but statistically significant increase in the misreading rate (Fig. 6C). Although we observed the tendency that the combined mutation of *rps31∆N* and *rps15*-1-141 further increased the misincorporation rate compared to the single mutations, while the combination of *rps31∆N* and *rps15*-F138S reduced the misreading rate compared to *rps31∆N* alone, no significance was detected for these combinations compared to the single mutations. Similarly, single *rps15* mutants F138S, F102L/F138S, and RTH(77–79)>A repeatedly showed slightly increased misreading rates in our assays, however without significance. In contrast, no difference compared to the wild-type was observed for the *rps15*-1-134 mutant. Interestingly, the *∆ltv1* knockout mutation also significantly increased the translation error rate compared to the wild-type strain, but only tendencies for increased misreading without significance were detected for the double *∆ltv1 rps15*-1-141 mutant, and for decreased misreading in the double *∆ltv1 rps15*-F138S mutant. Altogether, these results suggest that slight alterations in the C-terminal tail of Rps15 can lead to increased misreading. Moreover, our data support a model where the N-terminal tail of Rps31 and the tip of the C-terminal end of Rps15 (missing in the *rps15*-1-141 mutant) cooperate in ensuring translational fidelity. Ltv1 is a crucial factor for final maturation of cytoplasmic pre-40S particles, including correct positioning of different r-proteins [20,21,24]; thus, as our results showed, it is not surprising that 40S subunits are also prone to make more misreading in the absence of Ltv1.

## Discussion

Many eukaryotic r-proteins contain, in addition to globular domains, long unstructured N- or C-terminal extensions [50]. Interestingly, in several instances, such extensions were described to fulfil important functions during ribosome biogenesis. For example, the C-terminal tails of Rps14 and Rps5 and the N-terminal tail of Rps31 were shown to be required for 20S pre-rRNA processing in the cytoplasm [34,35,48]. Interestingly however, Rps5, Rps14 and Rps31 are assembled to pre-ribosomes early on, and the absence of the essential Rps5 and Rps14 r-proteins stalls the ribosome biogenesis pathway in the nucleus [31]. Hence, while their physical presence is required early in maturation, these r-proteins fulfil additional important functions in late 40S subunit maturation steps. Here, we show that Rps15 is yet another r-protein with more than one function in ribosome biogenesis, being not only involved in early nucleolar maturation events and required for 40S export, as previously reported [11,12,31,36], but also in cytoplasmic pre-40S maturation.

In particular, the mutant in which the seven C-terminal amino-acids of Rps15 are missing, *rps15*-1-134, accumulated 20S pre-rRNA in the cytoplasm, suggesting a defect in the final 40S maturation steps prior to D-site cleavage. The molecular reason for this phenotype is not yet clear, however, we consider it unlikely that the Rps15 C-terminal tail directly regulates 20S pre-rRNA processing for several reasons: (1) Nob1 is present in pre-40S particles purified from the *rps15*-1-134 mutant, excluding a defect in Nob1 recruitment. (2) Our *rps15* mutants did not exacerbate the mild slow-growth phenotype of a *NOB1*-TAP strain (data not shown), in contrast to other mutants more directly connected to D-site cleavage, like *rpl3*-W255C [27]. (3) In pre-40S structures, the Rps15 C-terminal tail is ~50 Å apart from Nob1 and more than 80 Å apart from the 3’ end of the 18S rRNA [29,45,51], making a direct engagement of the Rps15 C-terminal tail in D-site cleavage unlikely.

Notably, the Rps15 C-terminal tail alters its positioning throughout the translational cycle. In the post-decoding pre-translocation state of translation elongation, it is wedged between the A- and P-site tRNAs [41]. In line with this, deletion of the last 15 residues from the C-terminal tail of human RPS15 leads to defects in translation elongation, as was concluded from the reduction of polysomes and the accumulation of 80S particles [39]. Moreover, some of the mutations in the RPS15 C-terminus linked to CLL showed reduced overall translation, while others showed increased misreading rates or increased stop-codon readthrough (Figure S2C) [43].

Here, we show that the deletion of the very C-terminal residue of Rps15 (K141) results in increased misreading rates. This result is in compliance with structural data suggesting that this terminal lysine residue (K145 in human RPS15) is in direct contact with mRNA during decoding [41]. Moreover, the genetic interaction between *rps15* and *rps31* mutants, as well as the enhanced misreading rates in the *rps31*ΔN *rps15-1-141* double mutant suggest that the Rps31 N-terminal extension and the Rps15 C-terminal tail might act in concert in ensuring not only the correct execution of final pre-40S maturation events but also correct decoding. Interestingly, *RPS31* is non-essential, and also our *rps15*- mutants are viable, indicating that although these mutants display growth defects, they tolerate a certain extent of translational error. Consistently, the misreading rates we detected were quite modest, especially when compared to the values obtained for mutants of other r-proteins (e.g. *rps9B*-D94N) or the wild-type strain treated with low doses of paromomycin [49]. Moreover, we did not observe increased misreading in the *rps15*-1-134 mutant, which showed the strongest defects in cytoplasmic pre-40S maturation. Together with the previous results that only few of the human RPS15 C-terminal tail mutants show increased misreading (Figure S2C) [43], these data suggest that mild positional alterations at some critical residues of the tail can alter translational fidelity, potentially due to altered communication with mRNA or tRNAs, while other mutations or the complete absence of this tail do not have such an effect. However, the main defect of C-terminal tail mutants is likely not the altered misreading, as it occurs only in some mutants and the increase in misreading is only subtle.

Strikingly, the strategic positioning of the Rps15 C-terminal tail between the A- and P-site tRNAs resembles the orientation of the Rps15 C-terminal tail in pre-40S particles, which is clamped between assembly factors Tsr1 and Rio2 instead of tRNA (compare PDB 6Y0G [41] and PDB 6Y7C [45], Figure 3F and 3E). Considering this positioning, we speculate that Rio2 and Tsr1 might quality-check for the integrity of the Rps15 C-terminal tail. In the absence of an interaction of the C-terminal tail with Rio2 and/or Tsr1, further maturation (including 20S pre-rRNA processing) and engagement of faulty pre-40S particles lacking the Rps15 C-terminal tail in translation might be prevented, resulting in the accumulation of these immature 40S particles in 80S(-like) particles. An alternative possibility would be that the *rps15*-1-134 mutant might cause an earlier cytoplasmic pre-40S maturation defect (e.g. an altered rRNA structure or protein positioning) and that not the absence of the Rps15 C-terminal tail directly, but the defect caused by its absence is responsible for the blocking of subsequent pre-40S maturation steps. Last but not least, it might be possible that the Rps15 C-terminal tail is itself part of a quality control mechanism to test the functionality of 40S subunits, and that in its absence, a particular quality control step cannot take place, consequently trapping pre-40S particles in an inactive form.

While the functional connection of the Rps15 C-terminal tail to Tsr1 can be explained by the structural data demonstrating a direct interaction, the precise connection of the Rps15 C-terminal tail to Ltv1 is more puzzling. Ltv1 interacts with Enp1 and Rps3 [19,20], which are both positioned in the head domain of the pre-40S particle, as is the globular domain of Rps15. So far, the flexible and poorly structured protein Ltv1 has only been partly resolved on pre-40S particles [17,18,45,52]. However, biochemical interaction data indicate a direct interaction between Rps15 and Ltv1 [53]. Moreover, a human pre-40S particle cryo-EM analysis succeeded in visualizing a C-terminal α-helix of Ltv1 that is in direct contact with the globular domain of Rps15 [29]. In lack of further structural information, it is not possible to judge whether additional contacts are formed between Ltv1 and Rps15 or whether Ltv1 even interacts with the Rps15 C-terminal domain directly. The observed defects in the absence of Ltv1 might either be a consequence of the missing interaction with the Rps15 globular domain (potentially leading to an altered positioning of Rps15) or might be due to the effect of the missing Ltv1 on other proteins linked to Rps15 like Rio2 or Tsr1. The third possibility we envisage is an RNA folding problem in these mutants. *∆ltv*1 mutants are cold-sensitive, and the genetic interaction between *LTV1* and *RPS15* is also most pronounced at low temperatures. Cold-sensitive phenotypes are frequently observed upon RNA folding problems [54,55], hence we speculate that an altered rRNA structure occurring in the absence of Ltv1 might be the reason for the genetic interactions with Rps15.

Evidence for a cytoplasmic pre-40S maturation function of Rps15 also comes from a recent study, in which several different mutants including *rps15* mutants were investigated for defects in the cytoplasmic release of Ltv1, Rio2 and Tsr1 from pre-40S particles [56]. Mutants in the C-terminal tail of Rps15 showed defects in the release of Rio2 from pre-40S particles in the mentioned study [56]. In our experiments, however, we did not see Rio2 release defects in *rps15* C-terminal mutants (**data not shown**). The reason for these divergent results could be related to the fact that, in the study by Huang *et al*. [55], the phenotypes were investigated in a strain depleted for Fap7, a condition normally leading to the accumulation of 80S-like particles [25,57]. Hence, Rio2 release defects might only be visible when *rps15* mutations are combined with the depletion of Fap7.

In recent years, a strong connection between mutations clustering in the evolutionary-conserved C-terminal tail of human RPS15 (131-PGIGATHSSR-140) and aggressive, chemo-refractory CLL has been uncovered, and the molecular reason has been attributed to translational defects [42–44]. Although a mutation of F141, the residue corresponding to yeast F138, has not yet been observed in this context, F141 is positioned directly after a cluster of amino-acids found to be mutated in CLL (Figure S2B). Additionally, C-terminal truncation mutants tested in our study lack at least one (*rps15*-1-141), or several (*rps15*-1-134) of the equivalent CLL-linked residues. In human cells, it was shown that the respective mutated RPS15 variants are incorporated into ribosomes and negatively affect translation fidelity and global protein synthesis (Figure S3C) [43]. Our study revealed that in yeast, Rps15 C-terminal truncation mutants already show defects in the course of 40S subunit maturation before 40S particles are even joined with 60S subunits forming translation-competent ribosomes. Thus, a plausible alternative possibility to explain the disease relapse could be related to the 40S biogenesis defects, likely at the late cytoplasmic maturation steps and, therefore, should be considered in the future.

## Materials and Methods

### Yeast strains and genetic methods

The *S. cerevisiae* strains used in this study are W303 derivatives generated by integration at the genomic locus and are listed in **Table S1**. Yeast plasmids were constructed using standard recombinant DNA techniques and are listed in **Table S2**. All DNA fragments amplified by PCR were verified by sequencing. The *tsr1-1* and *tsr1-2* mutants were generated by random PCR mutagenesis as described previously [58,59].

### Identification of mutants from the SL screen

Mutants showing synthetic growth defects or synthetic lethality in combination with Δ*ltv1* were previously generated in a synthetic lethality (SL) screen [30]. The screen is based on a combination of the *ade2/ade3* red/white colony-sectoring assay and counter-selection on 5-FOA (5-fluoro orotic acid, Thermo Scientific)-containing plates and scores for the inability to lose a plasmid carrying an *LTV1* wild-type copy, resulting in a red non-sectoring, 5-FOA-sensitive phenotype (for more details, see [60]). The seven mutants that were not further characterized in the previous study [30] were first transformed with *LEU2*-plasmids containing genes already known to be genetically linked to *LTV1* (i.e. *PRP43, PFA1, NOB1* and *RIO2*). While mutants remained red upon transformation with non-complementing plasmids, the mutants carrying complementing plasmids recovered the ability to lose the *LTV1* wild-type plasmid, and hence showed red/white sectoring colonies. Using this strategy, we identified six of the seven mutants to be complemented by *RIO2*. Sequencing of the chromosomal *RIO2* copy confirmed mutation of this gene in these six mutants. The remaining SL mutant (#432) was transformed with a genomic *LEU2*-plasmid based library. Colonies showing a red/white sectoring phenotype were re-streaked onto plates lacking leucine (SDC-leu) and then on 5-FOA containing plates. Complementing plasmids resulting in red/white sectoring colonies on SDC-leu and growth on 5-FOA were isolated, and *LTV1* containing plasmids were identified by PCR. All other plasmids were subjected to DNA sequencing, revealing that all of them contained *RPS15*. Mutation of *RPS15* in the SL mutant #432 was subsequently confirmed by DNA sequencing at the genomic locus.

### Plasmid shuffle assays

The Δ*rps15* shuffle strain (Δ*rps15* [pRS316*-RPS15*]) was constructed by chromosomal deletion of *RPS15* in a diploid yeast strain, followed by transformation with the *URA3*-plasmid [pRS316-*RPS15*]. After tetrad dissection, the spores harbouring the gene knockout and the complementing *URA3* plasmid were recovered. A similar strategy was used to generate the *RPS15* shuffle Δ*ltv1* strain (Δ*rps15* Δ*ltv1* [pRS316*-RPS15*]).

The ‘double shuffle’ strain Δ*rps15* Δ*tsr1* [pRS316-*RPS15*] [pRS316-*TSR1*] was generated by crossing of the respective single shuffle strains and subsequent sporulation.

The *rps31*∆N-HA ∆*rps15* [pRS316-*RPS15*] shuffle strain was generated by crossing of the *∆rps15* [pRS316-*RPS15*] shuffle strain with a *rps31*∆N-HA strain (also known as *ubi3*∆N-HA [48]). The resulting diploid was sporulated, tetrads were dissected and a representative strain harbouring the appropriate alleles selected.

To analyse the growth phenotypes conferred by mutant alleles of *RPS15* either alone or in combination with Δ*ltv1* and *tsr1-* or *rps31-*mutations, we transformed the strains with the respective plasmids. Thereafter, transformants were spotted in 10-fold serial dilutions on 5-FOA containing plates to evaluate the phenotypes caused after loss of the *URA3-RPS15* plasmid or both the *URA3-RPS15* and *URA3-TSR1* plasmids. Subsequently, strains that were viable on 5-FOA containing plates were re-streaked on plates selecting for the transformed plasmids. Subsequently, these strains were spotted in 10-fold serial dilutions onto the respective plates and incubated at different temperatures.

### Fluorescence in situ hybridization (FISH), fluorescence microscopy

For fluorescence *in situ* hybridization, cells were grown in 50 ml SDC-leu medium at 30°C to an OD_600_ of ~0.5 and fixated with formaldehyde with a final concentration of 4% for 1 h at room temperature. After fixation, cells were washed twice with buffer containing 0.1 M KPO_4_ buffer (K_2_HPO_4_ and KH_2_PO_4_ mixed in the appropriate ratio to obtain a pH of 6.4) and washed once with washing buffer containing 0.1 M KPO_4_ and 1.2 M sorbitol (pH of 6.4). For cell wall lysis, cells were incubated with 1 ml washing buffer containing 500 µg/ml Zymolyase 100 T (Amsbio) for 60 min at room temperature, followed by one washing step with the washing buffer. Finally, the spheroplasts were resuspended in ~1.5-fold pellet volume, applied to adhesive coated 10-well diagnostic microscope slides (Thermo Scientific, LOT #381,613) and incubated for 10 min. For equilibration and to remove non-adhering cells by aspiration, spheroplasts were washed with a 2x SSC buffer (pH 7) and, afterwards, incubated in a humid chamber overnight at 37°C with hybridization buffer containing 50% formamide, 10% dextran sulphate sodium salt from *Leuconostoc ssp*. (Fluka), 125 µg/ml *E. coli* MRE600 tRNA (Boehring Mannheim GmbH), 500 µg/ml salmon sperm DNA sodium salt (AppliChem), 4x SSC, 1x Denhardt solution (Invitrogen) and approximately 0.8  pmoles of a Cy3-labelled ITS1-specific probe (5′-Cy3-ATGCTCTTGCCAAAACAAAAAAATCCATTTTCAAAATTATTAAATTTCTT-3′). After probing, spheroplasts were washed once with 200 ml 2x SSC, with 1x SSC and, finally, incubated with 200 ml 0.5x SSC containing 5 µg DAPI. After nuclear staining with DAPI, spheroplasts were washed twice with 0.5x SSC and the wells were dried and layered with Mowiol before the microscopy slides were covered with coverslips. Cells were imaged by fluorescence microscopy using either an Imager Z1 microscope (Carl Zeiss) with Plan-Apo-Chromat 100 oil immersion lens and a DICIII, 4’,6-diamidino-2-phenylindole, and a HECy3 filter, or a Leica DM6 B microscope, equipped with a DFC 9000 GT camera, using the PLAN APO 100x objective and narrow band TXR and LDA filters and the LasX software.

### Sucrose gradient analysis

Cells were grown in 70 ml SDC-leu medium at 30°C to an OD_600_ of ~0.5–0.7 (log-phase). Cycloheximide (CHX) was added to 50 ml culture in a final concentration of 100 µg/ml, and cells were incubated on ice for 5 min. After harvesting, cells were resuspended in lysis buffer containing 10 mM HCl-Tris (pH 7.5), 100 mM NaCl, 30 mM MgCl_2_ and 100 µg/ml CHX. After mechanical cell lysis using glass beads, 7 A_260_ units of the cell extracts were loaded onto 5–35% sucrose gradients containing 50 mM HCl-Tris (pH 7.5), 50 mM NaCl and 10 mM MgCl_2_ and centrifuged at 38,000 rpm at 4°C for 2 h 45 min using a Beckman Optima^TM^ LE-80 K Ultracentrifuge. Gradients were analysed using a UA-6 system (Teledyne Isco) with continuous monitoring at A_254_ nm.

### Northern blotting

For the analysis of polysome profile fractions by northern blotting, RNA from the fractions (approximately 500 µl) was extracted by mixing three times with phenol-chloroform-isoamyl alcohol (25:24:1) and once with chloroform-isoamyl alcohol (24:1). RNA was precipitated as described below.

For the analysis of total RNA by northern blotting, cells were grown in 50 ml SDC-leu medium at 30°C to an OD_600_ of 0.1–0.2 and shifted for 3 h to 25°C, 30°C or 37°C, respectively. Cells were resuspended in 200 µl lysis buffer containing 10 mM HCl-Tris (pH 7.5), 10 mM EDTA, and 0.5% SDS and mechanically lysed with 200 µl glass beads (0.5 mm diameter) for 3 min. After the addition of 450 µl lysis buffer, RNA was extracted by adding four times phenol-chloroform-isoamyl alcohol (25:24:1) and once chloroform-isoamyl alcohol (24:1) to the samples. RNA was precipitated by the addition of 1/10 volumes of 3 M sodium acetate (pH 5.2), 2.5 volumes of 100% ethanol and 1 µl GlycoBlue^TM^ co-precipitant (Invitrogen) and, after drying, dissolved in nuclease-free water. Three µg of the isolated RNA were separated on 1.6% MOPS-agarose gels containing 20 mM 3-(N-morpholino)-propanesulfonic acid (MOPS), 5 mM sodium acetate, 1 mM EDTA, 0.75% formaldehyde and ethidium bromide (pH 7.0), transferred overnight onto Hybond N^+^ nylon membranes (Amersham Biosciences) by capillary transfer and UV cross-linked to the membrane. Except for the probe E/C_2_ (27S A + B): 5′-GGCCAGCAATTTCAAGTTA-3′, which was hybridized at 37°C, hybridization with the following 5′-^32^P-radiolabeled oligonucleotides was performed at 42°C overnight in buffer containing 0.5 M Na_2_HPO_4_, pH 7.2, 7% SDS, and 1 mM EDTA: probe D/A_2_ (20S/21S): 5′-GACTCTCCATCTCTTGTCTTCTTG-3′, probe A_2_A_3_ (35S, 32S/33S, 27SA2, 23S, 21S): 5′-TGTTACCTCTGGGCCC-3′, probe 18S: 5′-GCATGGCTTAATCTTTGAGAC-3′, probe 25S: 5′-CTCCGCTTATTGATATGC-3′). After three subsequent washing steps with a buffer containing 40 mM Na_2_HPO_4_, pH 7.2, 1% SDS, signals were detected by exposing X-ray films. Membranes were regenerated by washing in 1% SDS prior to hybridization.

## Tsr1-TAP purification

The Tsr1-TAP *Δrps1*5 strains carrying the *LEU2*-plasmids expressing *RPS15, rps15*-1-134 or *rps15*-RTH/77-79)>A, respectively, were grown at 30°C in 4 l YPD each to an OD_600_ of 2. TAP purifications were performed in a lysis buffer containing 50 mM Tris–HCl (pH 7.5), 100 mM NaCl, 1.5 mM MgCl_2_, 0.075% NP-40 and 1 mM dithiothreitol (DTT). Prior to use, 1× Protease Inhibitor Mix FY (Serva) was added freshly to the lysis buffer. Cells were lysed by mechanical disruption using glass beads and the lysate was incubated with 300 µL IgG Sepharose™ 6 Fast Flow (GE Healthcare) at 4°C for 60 min. After incubation, beads were transferred into Mobicol columns (MoBiTec) and washed with buffer. Elution from IgG Sepharose™ beads was performed *via* TEV protease under rotation at room temperature for 70 min. A final concentration of 2 mM CaCl_2_ was added to the TEV eluates and they were then incubated with 300 µL Calmodulin Sepharose™ 4B (GE Healthcare) at 4°C for 60 min, washed with lysis buffer containing 2 mM CaCl_2_ and finally eluted with 5 mM EGTA. Protein samples were TCA-precipitated and dissolved in an SDS sample buffer, separated on NuPAGE™ 4–12% Bis–Tris gels (Invitrogen) and analysed via western blotting.

## Western blotting

Western blot analysis was performed using the following antibodies: α-CBP antibody (1:5000; Merck–Millipore, cat. no. 07–482), α-Rps3 antibody (1:50,000; provided by Matthias Seedorf), α-Nob1 antibody (1:3000; provided by David Tollervey), horseradish peroxidase conjugated α-HA antibody (1:5000; Roche, cat. no. 12,013,819,001), α-GAPDH antibody (1:40,000; Cell Signalling Technology, cat. no. 2118S), secondary α-rabbit horseradish peroxidase-conjugated antibody (1:15,000; Sigma, cat. no. A0545). Protein signals were visualized using the Clarity™ Western ECL Substrate Kit (Bio-Rad) and captured by Chemi-Doc™ Touch Imaging System (Bio-Rad).

### Quantification of translation accuracy

To measure the rate of amino acid misincorporation, the appropriate strains were transformed with a dual-luciferase reporter plasmid generously provided by David M. Bedwell (see **Table S2**). Luciferase activities were measured as previously described [49] using the Dual-Glo® Luciferase Assay System (Promega). Cells from each strain were grown in liquid SDC-ura medium to mid-log phase at 30°C and firefly and *Renilla* luciferase luminescence levels were measured at room temperature with a CLARIOstar 1.20 microplate reader (BMG Labtech, Germany) adjusted to endpoint read-type and default settings. Assays were repeated four times (biological replicas), each of these was replicated three times (technical replicas), and the data were expressed as the mean ± the standard deviation. Error rates for each strain were calculated as the percentage of the firefly/*Renilla* luciferase activity (mutant plasmid) divided by the firefly/*Renilla* luciferase activity (wild-type plasmid).

## Supplementary Material

Supplemental Material

## Notes

1. Recently, a new nomenclature for ribosomal proteins was introduced (Ban et al., 2014). In this publication, the standard nomenclature is used, and the new nomenclature is additionally indicated upon first mentioning of a r-protein.
