# Supplementary material for: The C-terminal tail of ribosomal protein Rps15 is engaged in cytoplasmic pre-40S maturation
Source: RNA Biol. 2022 Apr 19;19(1):560–74. doi: 10.1080/15476286.2022.2064073 (PMC9037480; doi:10.1080/15476286.2022.2064073)

LEGENDS TO THE SUPPLEMENTARY FIGURES

Supplementary Figure S1. Simplified overview of the yeast pre-rRNA processing steps and appearance of 23S and 21S pre-rRNAs upon rRNA maturation defects. The precursor 35S pre-rRNA, encoding for the mature 18S, 5.8S and 25S rRNA, is displayed on top, indicating all pre-rRNA cleavage sites. Focusing on 40S subunit maturation, the rRNA maturation steps of the 5.8S and 25S are only displayed in an abridged version. Delay or inhibition of pre-rRNA processing steps lead to aberrant products such as the 23S RNA, where the cleavages at sites A_0, A_1 and A_2 are skipped and processing occurs at site A_3, or the 21S RNA, where only cleavage at A_2 is skipped and cleavage occurs at site A_3.

Supplementary Figure S2. Sequence alignment of the highly conserved Rps15 r-protein. (A) Sequences were aligned with Clustal Omega and viewed in Jalview. (B) The sequences of yeast Rps15 and human RPS15 were aligned with Clustal Omega and viewed in Jalview. Mutations and truncations of Rps15 used in this study are indicated with a red asterisk or the following symbol, ⊦, respectively. RPS15 exchanges found in chronic lymphocytic leukemia (CLL) patients are marked with a red dot. (C) Only the C-terminal residues from the alignment in (B) are shown and the types of defects observed for the respective mutations are indicated.

Supplementary Figure S3. The Rps15-F138S protein is expressed normally. (A) To determine expression levels, crude extracts of chromosomally 2xHA-tagged *RPS15* and *rps15*-F138S carrying wild type *LTV1* or Δ*ltv1*, respectively, were analyzed via western blotting using the antibodies α-GAPDH (loading control) and α-HA (2xHA-*RPS15*). (B) Chromosomally HA-tagged *RPS15* and *rps15*-F138S variants carrying wild type *LTV1* or Δ*ltv1*, respectively, were spotted onto YPD plates and incubated at the indicated temperatures for 2 days. For comparison, also the wild-type *LTV1* and the Δ*ltv1* knockout strain were spotted.

Supplementary Figure S4. The *rps15*-F138S Δ*ltv1* mutant does not show a pre-40S export defect. Fluorescence microscopy of the strain Δ*rps15* (*LTV1)* and the double knock-out strain Δ*rps15* Δ*ltv1*, containing both a *LEU2*-*RPS3*-GFP plasmid as well as a *TRP1*-*RPS15* or *TRP1*-*rps15*-F138S plasmid, revealed that the synthetic lethality of *rps15*-F138S Δ*ltv1* (displayed in **Figure 3B**) is not caused by pre-40S export defects.

Supplementary Figure S5. Synthetic enhancement or lethality of *rps15*-F138S and *rps15*-F102L/138S mutants with distinct *ltv1*, *rio2* and *tsr1* alleles. (A) The *ltv1* SS>A mutant showing only cytoplasmic pre-40S maturation defects is genetically linked to *rps15* variants. A Δ*rps15* Δ*ltv1* strain complemented by *LTV1* or *ltv1*-S339A/S342A (SS>A) and *RPS15, rps15*-F138S or the double mutant *rps15*-F102L/F138S, respectively, were spotted on SDC-leu-trp plates and were incubated at the indicated temperatures for 4 days. (B) *RPS15* is not genetically linked to *PFA1*. The strain Δ*rps15* Δ*pfa1* was complemented by *LEU2-RPS15* or *LEU2-rps15*-F138S plasmids, respectively, either in combination with a *HIS3-PFA1* plasmid or with *HIS3*-empty plasmid (Δ*pfa1*), spotted on SDC-his-leu plates and incubated at the indicated temperatures for 4 days. (C) *rps15*-F138S is not genetically linked to *rio2-1*. The Δ*rps15* Δ*rio2* double shuffle strain containing the wild-type plasmids *URA3-RPS15* and *URA3-RIO2* was transformed with *HIS3-RPS15* plasmid or the variant plasmid *HIS3-rps15*-F138S as well as *LEU2-RIO2* or *LEU2-rio2-1* (Y73H, I186V, Y210H, E364D, D371G and D417G) plasmid, respectively. These strains were spotted onto SDC-leu-his plates as well as onto 5-FOA containing plates and were incubated at 23°C and 30°C for 3 days. (D) Genetic interaction between *RPS15* and *TSR1*. The Δ*rps15* Δ*tsr1* double shuffle strain carrying the wild-type plasmids *URA3-RPS15* and *URA3-TSR1* was transformed with plasmids *HIS3-RPS15*, *HIS3-rps15*-F138S or *HIS3-rps15*-F102L/F138S as well as *LEU2-TSR1* or *LEU2-tsr1-1* (E588D, Y604C, T704A) or *LEU2-tsr1-2* (F220L, L239F, R473G, E481G, F596Y, F710L, T716A, F759L) plasmid, respectively. Strains were spotted on SDC-his-leu plates and 5-FOA containing plates and incubated at 23°C and 30°C for 3 days. Note that **Figure S5D** is the same as **Figure 3G** except for the additional panel -his-leu at 30ºC.

Supplementary Figure S6. Genetic interaction between *LTV1* and *RPS15*. (A and C) The Δ*rps15* (*LTV1*) and Δ*rps15* Δ*ltv1* double shuffle strain carrying *LEU2*-plasmids with *RPS15* variants were spotted on SDC-leu or 5-FOA containing plates, and were incubated at the indicated temperatures for 3 or 6 days as depicted. (B and D) To investigate the growth defects of all viable *rps15* mutants, the shuffled strains were spotted onto SDC-leu plates at different temperatures as indicated. (A and B) Amino acid exchanges of *RPS15* in close proximity to F102L are genetically linked to *LTV1*, except the mutated allele R77A. The *rps15* triple mutation R77A/T78A/H79A (RTH(77-79)>A) is lethal in combination with Δ*ltv1*, shown in A. Moreover, all other mutated *rps15* alleles, except R77A, revealed a growth defect in the Δ*ltv1* knock-out strain, especially at low temperatures, depicted in B. (C and D) C-terminal truncations of *RPS15* exhibit a growth defect, and deletion of the last amino acid of *RPS15*

already shows genetic interaction with *LTV1.* Deletion of the last eight amino acids of Rps15 leads to a strong growth defect on 5-FOA containing plates, which is even more enhanced when the last 12 amino acids are deleted, shown in C. On the other hand, these two *RPS15* mutants are lethal in the absence of *LTV1*. Truncations at the C-terminus leads to growth defects, especially at low and high temperatures, shown in D.

Supplementary Figure S7. The *rps15* -1-134 and *rps15*-RTH(77-79)>A mutants show no ribosomal subunit export defect. The Δ*rps15* strains containing *LEU2*-plasmids with *RPS15* alleles as indicated were transformed with *URA3*-Rps3-GFP or *URA3*-Rpl25-GFP small and large subunit export reporter plasmids, respectively. Cells were cultivated in SDC-ura-leu media and inspected in logarithmic phase by fluorescence microscopy.

Supplementary Figure S8. Genetic interaction between *RPS31* and *RPS15*. The Δ*rps15* (*RPS31*) and Δ*rps15 rps31*ΔN strains carrying *LEU2*-plasmids with *RPS15* variants were spotted onto YPD plates and incubated at 30°C for 4 days. Only *rps15*-1-141 *and rps15*-F138S were viable in combination with *rps31*ΔN (shown in **Figure 6B**), therefore only for these, double mutants could be tested.

Supplementary Tables

Supplementary Table 1. Yeast strains

name	genotype	source
W303	*ade2 leu2 trp1 ura3*	[1]
Δ*ltv1*	W303 *MAT***a** Δ*ade3::kanMX4* Δ*ltv1::kanMX6*	[2]
Δ*rps15* Δ*ltv1*	W303 *MAT***a** Δ*rps15*::kanMX4 Δ*ltv1*::natNT2 [pRS316-*RPS15*]	this study
Δ*rps15*	W303 *MAT***a** Δ*rps15*::kanMX [pRS316-*RPS15*]	this study
Δ*rps15* Δ*tsr1*	W303 *MAT***a** Δ*rps15*::kanMX Δ*tsr1*::*HIS3*MX [pRS316-*RPS15*] [pRS316-*TSR1*]	this study
Δ*rps15* Δ*pfa1*	W303 *MAT***a** Δ*rps15*::kanMX4 Δ*pfa1*::natNT2 [pRS316-*RPS15*]	this study
Δ*rps15* Δ*rio2*	*MAT***α** Δ*rps15*::kanMX Δ*rio2*::kanMX *his3 leu2 lys2 ura3 trp1* [pRS416-*RIO2*] [pRS316-*RPS15*]	this study
TLY63.A3 (*rps31*ΔN)	W303 *MAT***α** *ubi3*ΔN-HA::kanMX4	[3]
Δ*rps15* *rps31*ΔN	W303 *MAT***α** *ubi3ΔN-HA::kanMX4* Δ*rps15*::kanMX [pRS316-*RPS15*]	this study
HA-*RPS15*	W303 *MAT***a** P*RPS15* 2xHA::natNT2-*RPS15*	this study
HA-*rps15* F138S	W303 *MAT***a** P*RPS15* 2xHA::natNT2-*rps15* F138S	this study
Δ*ltv1* HA-*RPS15*	W303 *MAT***a** P*RPS15* 2xHA::natNT2-*RPS15* Δ*ltv1::hphNT1*	this study
Δ*ltv1* HA-*rps15* F138S	W303 *MAT***a** P*RPS15* 2xHA::natNT2-*rps15* F138S *hphNT1*	this study

Supplementary Table 2. ***S.cerevisiae*** **and** ***E.coli*** **plasmids.**

name	relevant information	source
pRS316-*RPS3*-eGFP	CEN, *URA3*, P*RPS3,* T*RPS3*	[4]
pRS316-*RPL25*-eGFP	CEN, *URA3*, P*RPL25,* T*RPL25*	[5]
pHT4467-ΔCEN-*LTV1*	*URA3*, *ADE3*, P*LTV1,* T*LTV1*	[2]
pRS314-*RPS15*	CEN, *TRP1*, P*RPS15,* T*RPS15*	this study
pRS314-*rps15* F138S	CEN, *TRP1*, P*RPS15,* T*RPS15*	this study
pRS314-*rps15* F102L F138S	CEN, *TRP1*, P*RPS15,* T*RPS15*	this study
pRS315 (empty vector)	CEN, *LEU2*	[6]
pRS315-*RIO2*	CEN, *LEU2*, P*RIO2,* T*RIO2*	[4,7]

pRS315-*rio2-1*	CEN, *LEU2*, P*RIO2,* T*RIO2*	[7]
pRS315-*TSR1*	CEN, *LEU2*, P*TSR1,* T*TSR1*	this study
pRS315-*tsr1-1*	CEN, *LEU2*, P*TSR1,* T*TSR1*	this study
pRS315-*tsr1-2*	CEN, *LEU2*, P*TSR1,* T*TSR1*	this study
pRS315-*LTV1*	CEN, *LEU2*, P*LTV1,* T*LTV1*	[8]
pRS315-*ltv1* SS>A	CEN, *LEU2*, P*LTV1,* T*LTV1*	[8]
pRS315-*RPS15*	CEN, *LEU2,* P*RPS15,* T*RPS15*	this study
pRS315-*rps15* F138S	CEN, *LEU2,* P*RPS15,* T*RPS15*	this study
pRS315-*rps15* F102L F138S	CEN, *LEU2,* P*RPS15,* T*RPS15*	this study
pRS315-*rps15* F102L	CEN, *LEU2,* P*RPS15,* T*RPS15*	this study
pRS315-*rps15* R77A	CEN, *LEU2,* P*RPS15,* T*RPS15*	this study
pRS315-*rps15* T78A	CEN, *LEU2,* P*RPS15,* T*RPS15*	this study
pRS315-*rps15* H79A	CEN, *LEU2,* P*RPS15,* T*RPS15*	this study
pRS315-*rps15* R77A H79A	CEN, *LEU2,* P*RPS15,* T*RPS15*	this study
pRS315-*rps15* RTH(77-79)>A	CEN, *LEU2,* P*RPS15,* T*RPS15*	this study
pRS315-*rps15* 1-141	CEN, *LEU2,* P*RPS15,* T*RPS15*	this study
pRS315-*rps15* 1-135	CEN, *LEU2,* P*RPS15,* T*RPS15*	this study
pRS315-*rps15* 1-134	CEN, *LEU2,* P*RPS15,* T*RPS15*	this study
pRS315-*rps15* 1-130	CEN, *LEU2,* P*RPS15,* T*RPS15*	this study
pRS313 (empty vector)	CEN, *HIS3*	[6]
pRS313-*PFA1*	CEN, *HIS3,* P*PFA1,* T*PFA1*	[2]
YEplac195-lucCAAA (pDB688)	Renilla and Firefly construct containing a CAC codon control (H245); *2μ, URA3*	[9]
YEplac195-lucCAAA H245 CGC (pDB868)	Renilla and Firefly construct containing a CGC codon (H245R); *2μ, URA3*	[9]
pRS316-*RPS15*	CEN, *URA3,* P*RPS15,* T*RPS15*	this study
pRS316-*RIO2*	CEN, *URA3,* P*RIO2,* T*RIO2*	[4]
pRS316-*TSR1*	CEN, *URA3,* P*TSR1,* T*TSR1*	this study
pFA6a-*HIS3*MX4	for chromosomal deletion	[10]
pFA6a-kanMX4	for chromosomal deletion	[10]
pFA6a-natNT2	for chromosomal deletion	[10,11]

References

1. Thomas BJ, Rothstein R. The genetic control of direct-repeat recombination in Saccharomyces: The effect of rad52 and rad1 on mitotic recombination at GAL10, a transcriptionally regulated gene. Genetics 1989:725–738.
2. Pertschy B, Schneider C, Gnädig M, et al. RNA helicase Prp43 and its co-factor Pfa1 promote 20 to 18S rRNA processing catalyzed by the endonuclease Nob1. J Biol Chem. 2009;284(50):35079–35091.
3. Fernández-Pevida A, Martín-Villanueva S, Murat G, et al. The eukaryote-specific N-terminal extension of ribosomal protein S31 contributes to the assembly and function of 40S ribosomal subunits. Nucleic Acids Res. 2016;44(16):7777–7791.
4. Schäfer T, Maco B, Petfalski E, et al. Hrr25-dependent phosphorylation state regulates organization of the pre-40S subunit. Nature 2006;441(7093):651–655.
5. Gadal O, Strauss D, Kessl J, et al. Nuclear export of 60s ribosomal subunits depends on Xpo1p and requires a nuclear export sequence-containing factor, Nmd3p, that associates with the large subunit protein Rpl10p. Mol Cell Biol. 2001;21(10):3405–3415.
6. Robert S. Sikorski and Philip Hieter, Sikorski RS, Hieter P. A system of shuttle vectors and yeast host strains designed for efficient manipulation of DNA in Saccharomyces cerevisiae. Genetics 1989;122(1):19–27.
7. Schäfer T, Strauss D, Petfalski E, et al. The path from nucleolar 90S to cytoplasmic 40S pre-ribosomes. EMBO J. 2003;22(6):1370–1380.
8. Mitterer V, Murat G, Réty S, et al. Sequential domain assembly of ribosomal protein S3 drives 40S subunit maturation. Nat Commun. 2016;7:10336.
9. Salas-Marco J, Bedwell DM. Discrimination between defects in elongation fidelity and termination efficiency provides mechanistic insights into translational readthrough. J Mol Biol. 2005;348(4):801–815.
10. Longtine MS, McKenzie A, Demarini DJ, et al. Additional modules for versatile and economical PCR-based gene deletion and modification in Saccharomyces cerevisiae. Yeast 1998;14(10):953–961.
11. Janke C, Magiera MM, Rathfelder N, et al. A versatile toolbox for PCR-based tagging of yeast genes: new fluorescent proteins, more markers and promoter substitution cassettes. Yeast 2004;21(11):947–962.

Supplementary figure 1

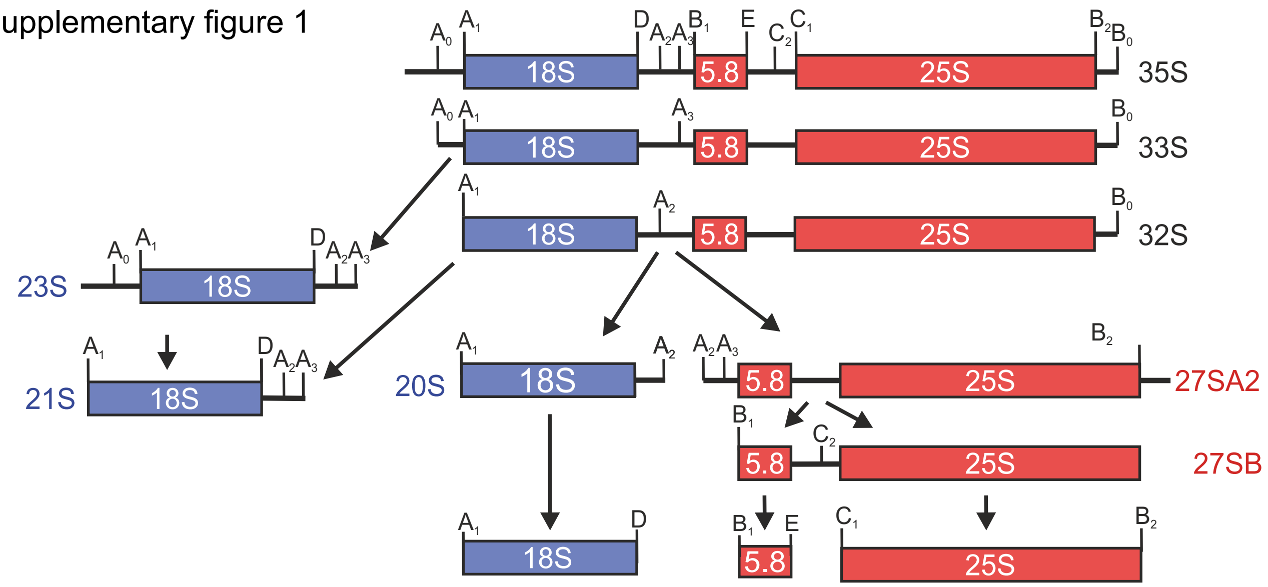

Supplementary figure 2

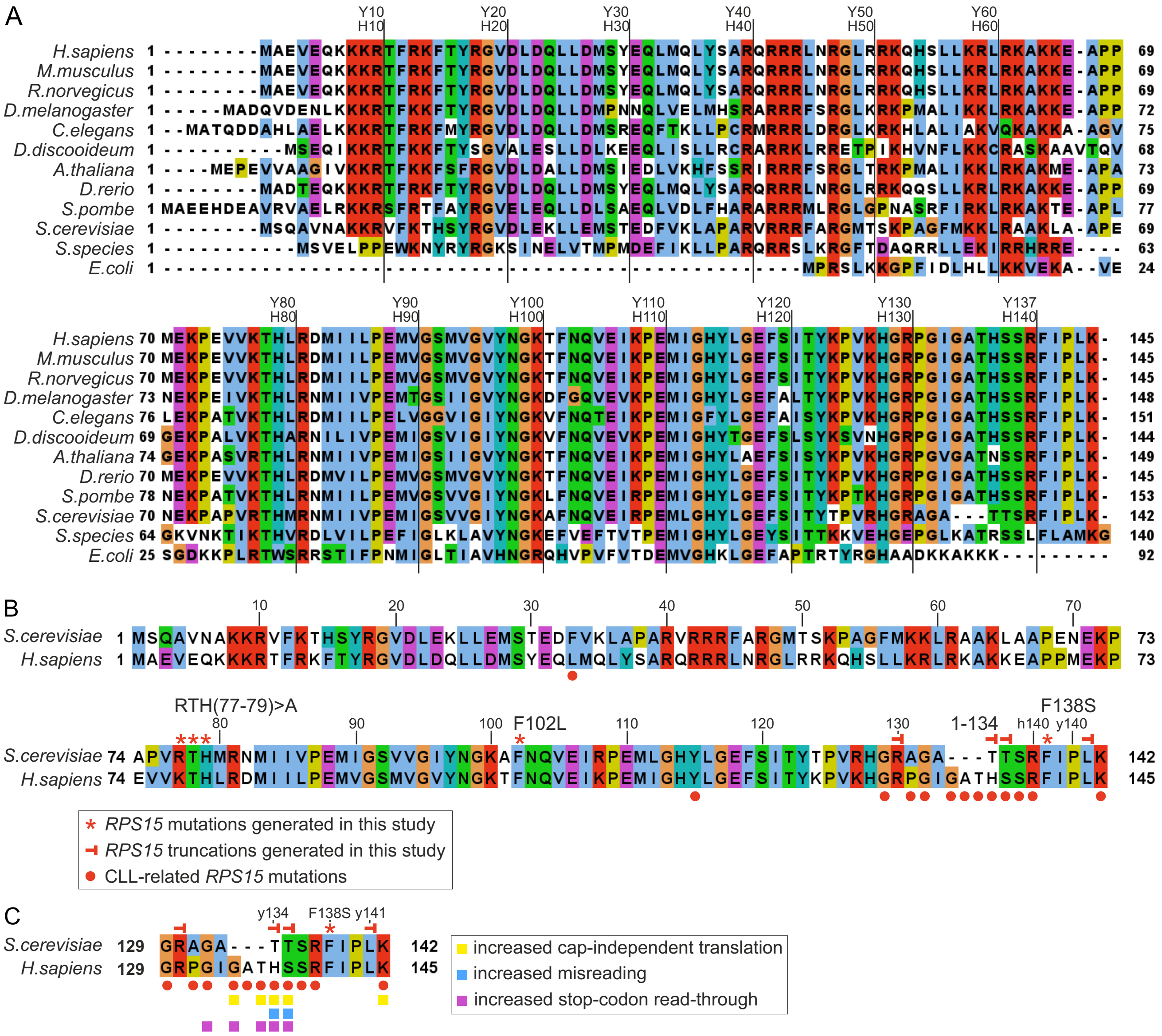

Supplementary figure 3

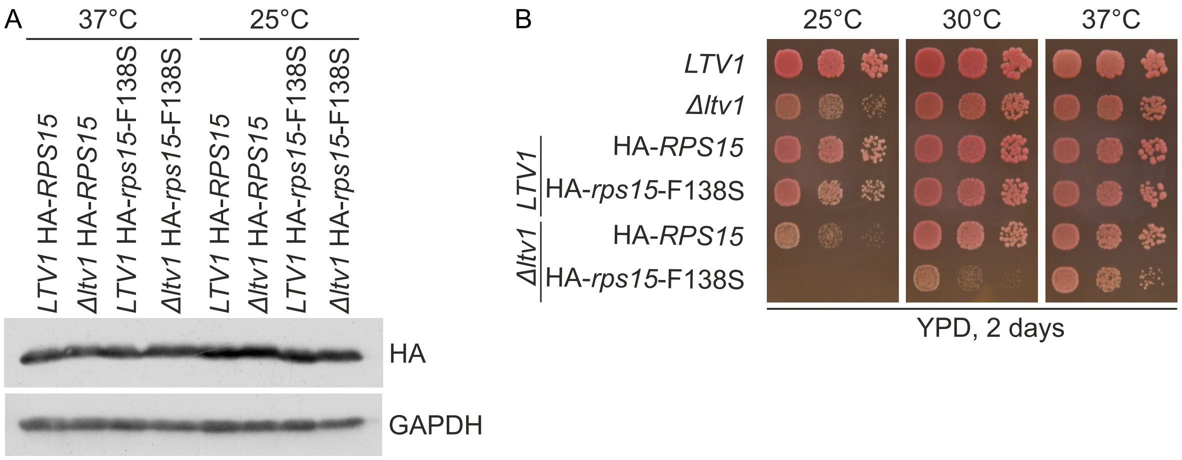

Supplementary figure 4

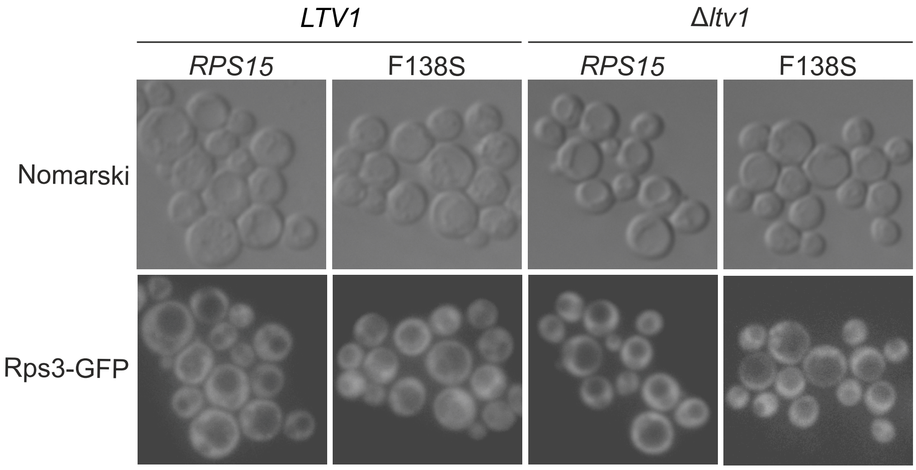

Supplementary figure 5

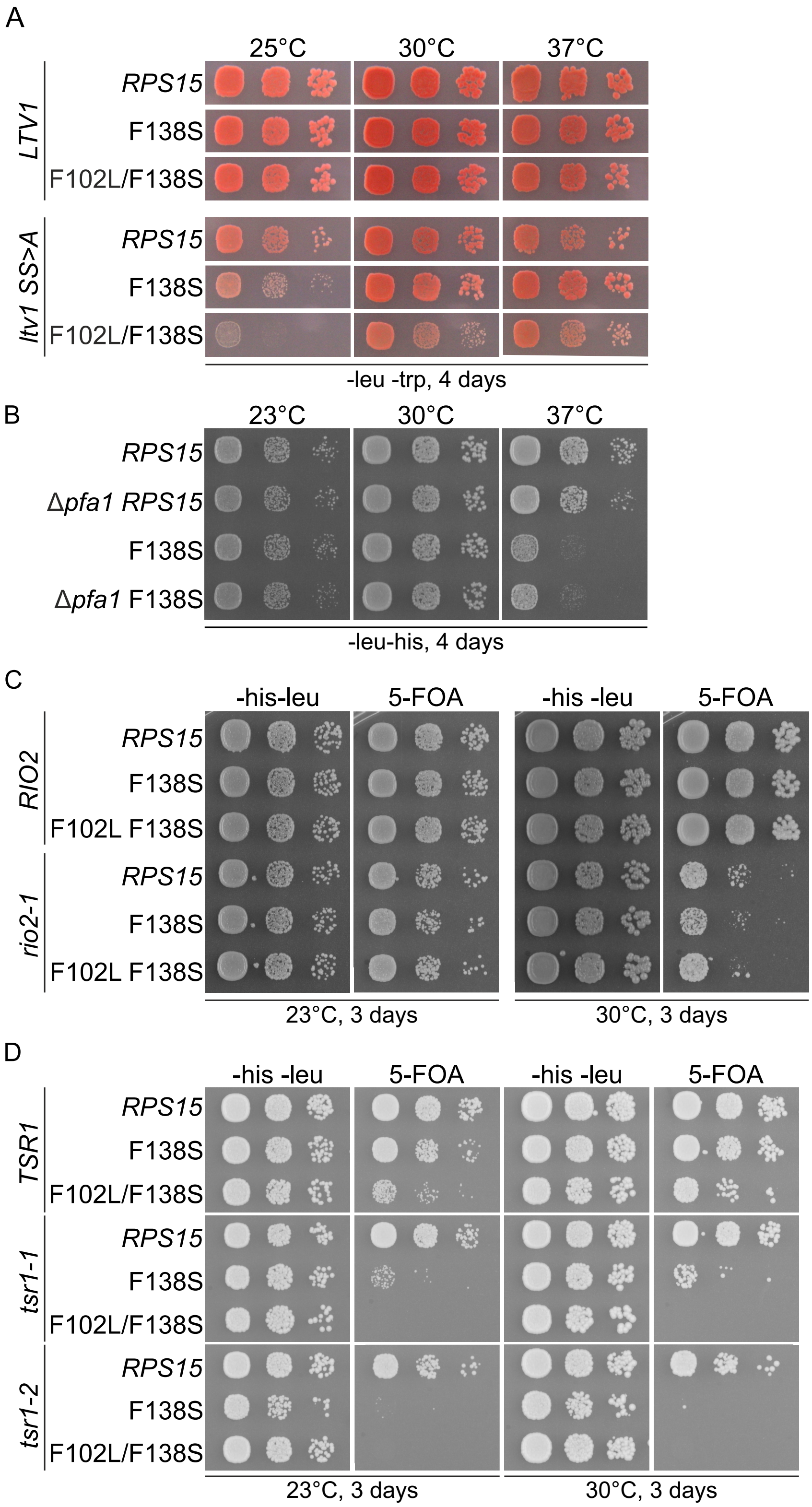

Supplementary figure 6

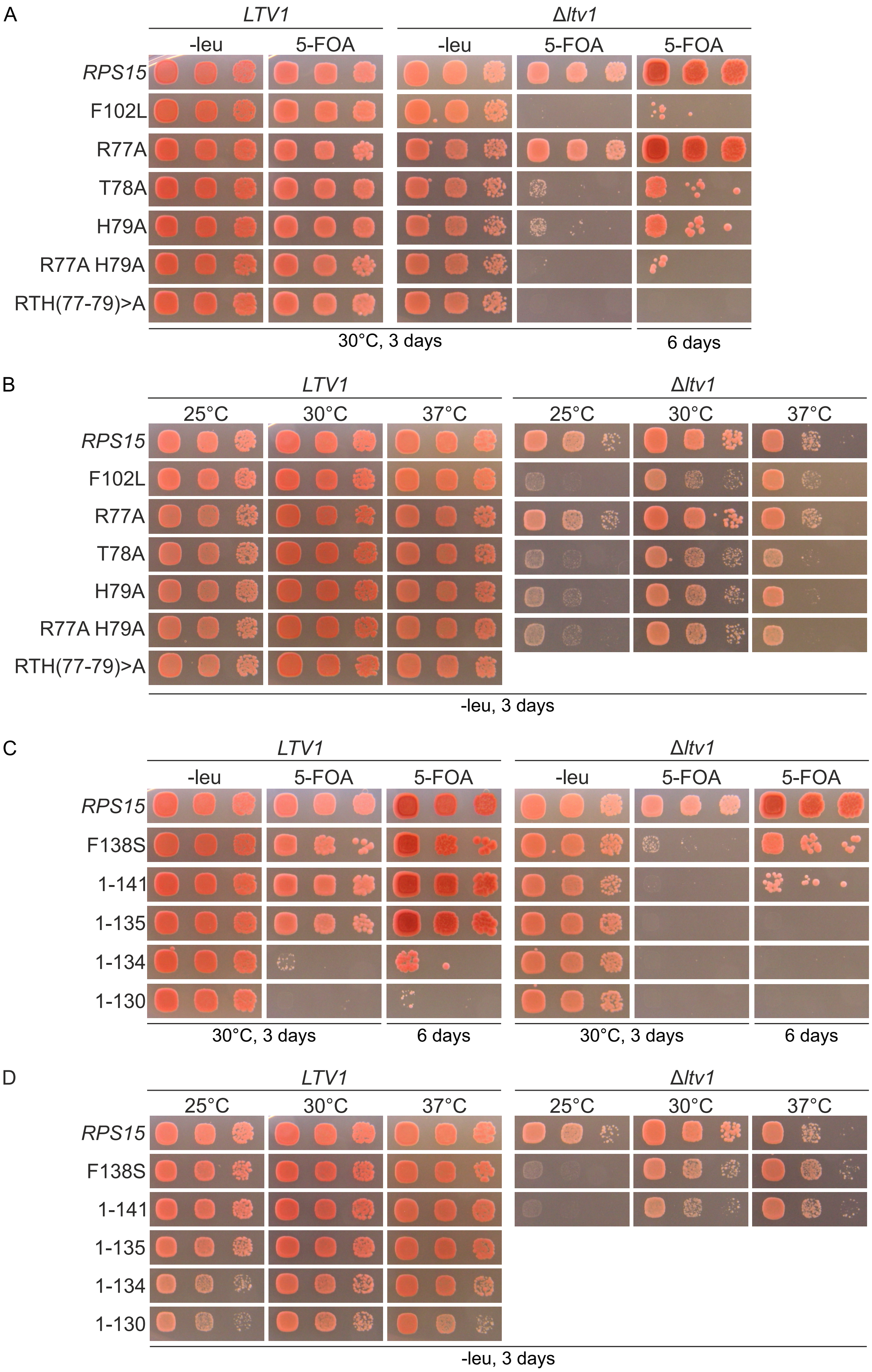

Supplementary figure 7

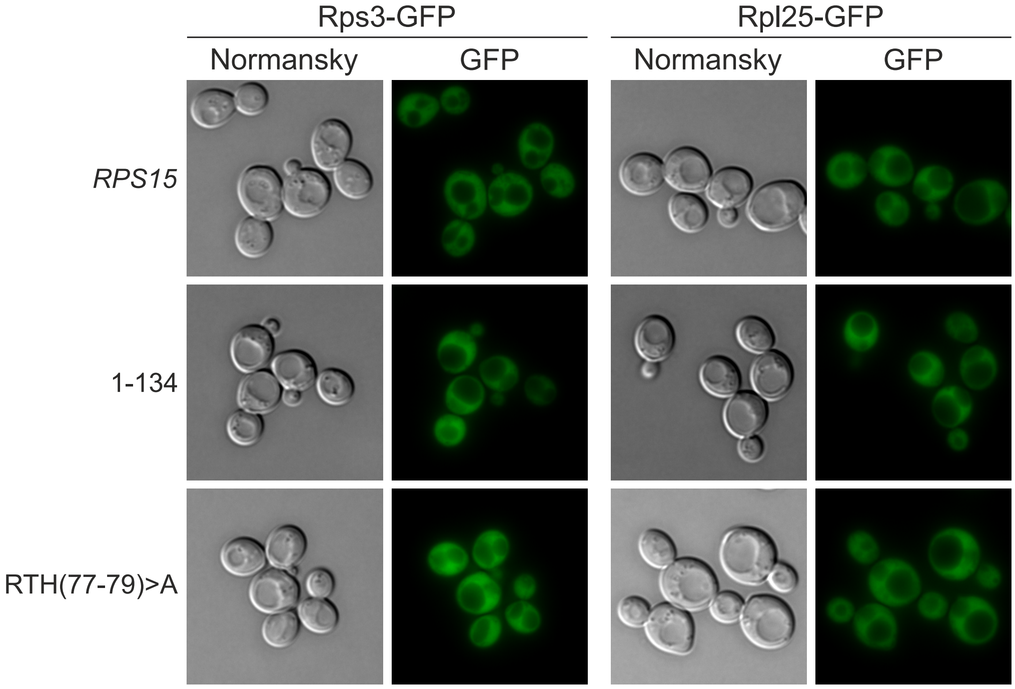

Supplementary figure 8

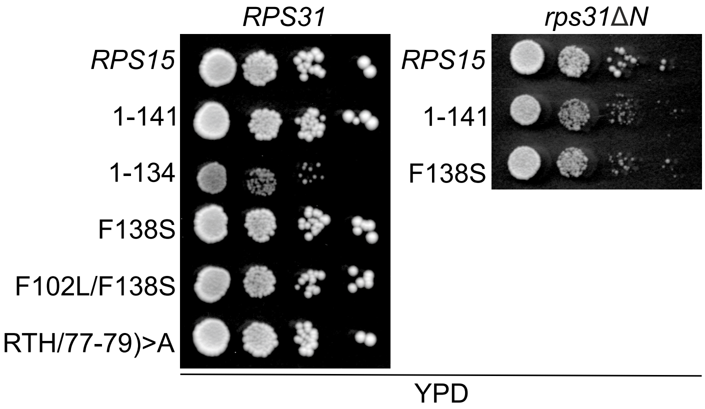

Supplement: Supplemental Material [file KRNB_A_2064073_SM5506.pdf]
